# A histidine-mediated supramolecular mechanism links temperature and pH activation of the sperm channel CatSper

**DOI:** 10.1371/journal.pbio.3003776

**Published:** 2026-10-09

**Authors:** Billy Zhao, Shweta Bhagwat, Juan Ferreira, Kiersten M. Ruff, Dilip K. Swain, Rohit V. Pappu, Celia M. Santi, Ziao Fu, Polina V. Lishko

**Affiliations:** 1 Department of Cell Biology and Physiology, Washington University School of Medicine in St. Louis, St. Louis, Missouri, United States of America; 2 Center for the Investigation of Membrane Excitability Diseases (CIMED), Washington University School of Medicine in St. Louis, St. Louis, Missouri, United States of America; 3 Department of Obstetrics and Gynecology, Washington University School of Medicine in St. Louis, St. Louis, Missouri, United States of America; 4 Department of Biomedical Engineering and Center for Biomolecular Condensates, McKelvey School of Engineering, Washington University in St. Louis, St. Louis, Missouri, United States of America; 5 BJC Investigator Program, Washington University School of Medicine in St. Louis, St. Louis, Missouri, United States of America; University of Zurich, SWITZERLAND

## Abstract

The cation channel of sperm, CatSper, is a sperm-specific calcium channel essential for male fertility across metazoans. CatSper is activated by intracellular alkalinization, membrane depolarization, and elevated temperature, which together ensure maximal current generation. However, the structural mechanisms by which these physiological stimuli jointly regulate channel activation remain poorly understood, in part because CatSper is an unusually complex ~15-subunit assembly organized in zigzag supramolecular arrays along the sperm flagellum. Here, we combine comparative genomics across 47 species with AlphaFold3-based modeling and evolutionary sequence-structure analyses to investigate a mechanism underlying temperature and pH sensitivity of CatSper. Phylogenetic analysis shows that N-terminal length and histidine enrichment of the pore-forming subunit CatSper1 strongly correlate with species-specific fertilization temperatures, suggesting evolutionary adaptation of temperature and pH sensitivity. Structural modeling further revealed conserved surface-exposed histidine clusters that form predicted contact interfaces between adjacent CatSper assemblies, with this contact interface positioned near the dominant voltage-sensing module. Functional validation using electrophysiology and calcium imaging in mouse sperm shows that capacitation-associated partial removal of the CatSper1 N-terminus selectively impairs temperature-dependent activation. Together, these findings support a model in which temperature-dependent histidine deprotonation functionally couples neighboring CatSper complexes within the supramolecular assembly, thereby promoting synchronized channel activation.

## Abstract 2

CatSper is a sperm-specific calcium channel essential for male fertility that is activated by changes in pH and temperature, but how these signals are integrated remains unclear. This study provides evidence suggesting that temperature-dependent histidine deprotonation within the CatSper1 N-terminus promotes neighboring complex coupling and activation.

## Introduction

The cation channel of sperm (CatSper) is a sperm-specific calcium ion channel that plays an important role in sperm function across the evolutionary tree and is essential for male fertility in mice and humans [1–15]. Originally identified in mice [1], CatSper channels are indispensable for hyperactivation—an asymmetric flagellar bending pattern required for successful fertilization—which is triggered by calcium influx through this ion channel [1,8,16]. Because calcium entry must be tightly controlled, CatSper activity is regulated by multiple modalities that ensure it functions only under precise physiological conditions. CatSper is gated by warm temperatures [17], membrane depolarization [6,12,18,19], and intracellular alkalinization [6,12,19], while cytoplasmic acidification potently inhibits its function [6].

This multimodal regulation is supported by the structural complexity of CatSper, which forms a large multiprotein complex composed of at least 15 distinct subunits, each encoded by a separate gene [1,4,7,13,17,20–32], and is called CatSpermasome [31]. Each CatSpermasome is further assembled into highly ordered zigzag arrays that form four longitudinal rows running along the entire length of the sperm principal piece [21].

Comparative genomic studies have revealed that while CatSper genes are broadly conserved across vertebrates, they exhibit striking lineage-specific patterns of gain and loss, highlighting the channel’s evolutionary diversity [10,15,20,33]. In mammals such as mice and humans, CatSper is indispensable for male fertility; however, in certain avian and fish lineages, as well as mollusks, the channel has been lost, suggesting the evolution of alternative mechanisms for sperm activation [10,15,34].

The pore of the CatSper channel is formed by the relatively homologous subunits CatSper1, CatSper2, CatSper3, and CatSper4 [7]. All four are six-transmembrane domain polypeptides encoded by separate genes, and they share a conserved selectivity filter. While most other CatSper subunits are conserved, one in particular- CatSper1 – shows exceptional divergence across species, particularly in the length of its N-terminal domain highly enriched in histidine residues.

The activity of CatSper complex is also time-dependent. As sperm undergo their final maturation within the female reproductive tract, a process known as capacitation [35,36], the composition of the plasma membrane changes and sperm proteins undergo multiple modifications, including tyrosine phosphorylation [37,38] and targeted proteolytic processing [39]. These changes progressively prime sperm for optimal function. However, excessive or prolonged capacitation can ultimately compromise sperm performance. For example, murine sperm that undergo capacitation for more than 90 min show reduced functional performance [39]. Interestingly, the CatSper complex undergoes capacitation-dependent proteolytic cleavage that specifically removed the portion of N-terminal domain of CatSper1 [14,39]. The timing of this cleavage closely coincides with the reported loss of CatSper temperature sensitivity [17], suggesting that the CatSper1 N-terminal domain may play a central role in temperature-dependent channel regulation.

To explain the multimodal regulation of CatSper, such as its ability to integrate pH and thermal signals to fine-tune calcium entry during sperm hyperactivation, we combined comparative genomics with AlphaFold3 [40] prediction. This analysis produced a mechanistic model for the operation of this unique ion channel complex. According to this model, surface-exposed histidine residues in the N-terminal domain of CatSper1 engage in pH-dependent interactions, stabilizing a previously presumed disordered region and promote coupling among adjacent CatSper channels. Increasing temperature would favor histidine deprotonation, thereby strengthening these interactions and enhancing coupling across CatSper channel array. This cooperative mechanism could promote synchronized channel opening and provide a framework for understanding how CatSper integrates pH and temperature signals to regulate Ca^2+^ influx during sperm activation.

The importance of CatSper1 N-terminal domain for temperature sensitivity was further supported by electrophysiological and calcium imaging recording from murine sperm that underwent partial degradation of the N-terminal domain [14,17,39].

## Results

### Comparative genomics analysis revealed a correlation between N-terminal composition of CatSper1 and optimal fertilization temperature

The core of the CatSpermasome complex is formed by four pore-forming subunits: CatSper1, CatSper2, CatSper3, and CatSper4 [7], while four auxiliary subunits- CatSper β [27], δ [13], ε [23,41], and γ [25]—extend large extracellular domains that form a canopy over the channel pore [21,31]. All eight CatSper subunits are transmembrane proteins and are largely conserved across species, with the notable exception of CatSper1, which exhibits substantial variation in length (Figs 1A–1B and S1A) and shows a positive correlation between its overall length and species-specific fertilization temperatures (Figs 1C and S1B). The feature that sets CatSper1 apart is its unusually long N-terminal domain (NTD), whose length also scales up with fertilization temperature and is highly enriched in histidine (His, H) residues (Fig 1D). Interestingly, marine invertebrates such as corals and sea urchins that spawn their sperm directly into sea water, feature truncated NTDs barely reaching 120 amino acids (Fig 1A–1D and Table 1). This trend was shared with reptiles that presented a slightly elongated NTD around 127 amino acids. As the core body temperature increased, particularly for internal fertilizers, the length of the domain rose exponentially, reaching ~500 residues in species such as *Lontra Canadensis* (River Otter) and *Oryctolagus Cuniculus* (European Rabbit).

**Table 1:** Species included in this study and their CatSper-related parameters. The lengths of the CatSper1 subunit and its N-terminal domain (NT) are reported as numbers of amino acid residues (aa). Additional columns include the theoretical isoelectric point (pI) of the CatSper1 N-terminus, fertilization temperature in °C, percentage (%) and absolute number of histidine residues (#) within the N-terminus, and the corresponding references for fertilization temperatures for each species. Colored area depicts cold-fertilizing species (6–23 °C; blue), intermediate temperatures (25–34.5 °C; green), and warm-fertilizing species (35–40 °C; red).

	Species, name	Common name	T, °C	NT, aa	His in NT		NT, pI	CatSper1 length, aa	Ref.
					%	#			
1	*Oncorhynchus mykiss*	Rainbow trout	7	65	4.6	3	10.7	424	[59]
2	*Salmo salar*	Atlantic salmon	6	111	3.6	4	10.1	470	[60]
3	*Lamellibrachia satsuma*	Satsuma tube worm	14	15	0	0	4.4	351	[61]
4	*Ciona intestinalis*	Sea squirt	15	89	4.5	4	9.8	465	[62]
5	*Strongylocentrotus purpuratus*	Purple sea urchin	15.5	120	5.8	7	6.7	474	[63]
6	*Apostichopus japonicus*	Japanese sea cucumber	16	102	2.9	3	10.1	456	[64]
7	*Nematostella vectensis*	Starlet sea anemone	20	11	9.1	1	11.2	385	[65]
8	*Paramuricea clavata*	Red gorgonian sea fan	23	9	33.3	3	11.0	230	[66]
9	*Orchesella cincta*	Springtail	25	191	2.6	5	5.3	633	[67]
10	*Stylophora pistillata*	Smooth cauliflower coral	25.5	12	16.7	2	9.7	340	[68]
11	*Lingula anatina*	Lingula brachiopod	26	138	3.6	5	6.5	496	[69]
12	*Acropora digitifera*	Finger coral	28	10	10	1	11.0	260	[70]
13	*Acropora millepora*	Staghorn coral	28	10	10	1	11.0	383	[71]
14	*Pelodiscus sinensis*	Chinese soft-shell turtle	28.5	95	1.1	1	10.0	493	[72]
15	*Alligator mississippiensis*	American alligator	31	102	2.0	2	11.2	430	[73]
16	*Heterocephalus glaber*	Naked mole-rat	31	459	26.4	121	6.8	816	[74]
17	*Ornithorhynchus anatinus*	Platypus	32.5	112	3.6	4	10.7	466	[75]
18	*Podarcis muralis*	Common wall lizard	33.5	127	3.2	4	11.9	488	[76]
19	*Vombatus ursinus*	Common wombat	34	74	4.1	3	8.9	428	[77]
20	*Monodelphis domestica*	Gray short-tailed opossum	34	107	3.7	4	9.9	468	[78]
21	*Varanus komodoensis*	Komodo dragon	34.5	124	3.2	4	11.8	626	[79]
22	*Elephantulus edwardii*	Cape Rock Elephant Shrew	35	226	5.8	13	10.9	580	[80]
23	*Sarcophilus harrisii*	Tasmanian devil	35.5	141	5.0	7	11.1	491	[81]
24	*Fukomys damarensis*	Damaraland mole-rat	35.5	556	26.4	147	6.8	953	[82]
25	*Phascolarctos cinereus*	Koala	36	113	6.2	7	10.1	467	[83]
26	*Lipotes vexillifer*	Yangtze river dolphin (baiji)	36	351	19.1	67	7.5	705	[84]
27	*Loxodonta africana*	African elephant	36	449	21.2	95	7.0	804	[85]
28	*Notamacropus eugenii*	Tammar wallaby	36	113	7.1	8	10.2	467	[86]
29	*Tursiops truncatus*	Bottlenose dolphin	36.5	192	7.3	14	8.0	547	[87]
30	*Homo sapiens*	Human	36.5	428	19.4	83	7.6	780	[88]
31	*Mus musculus*	Domestic mouse	36.5	332	17.2	57	8.2	686	[17,89]
32	*Macaca mulatta*	Rhesus macaque	37	413	18.9	78	7.4	798	[90]
33	*Mirounga angustirostris*	Northern elephant seal	37	374	15.0	56	8.6	727	[91]
34	*Physeter macrocephalus*	Sperm whale	37	265	15.5	41	9.4	619	[92]
35	*Myotis lucifugus*	Little brown bat	37	322	17.4	56	9.7	678	[93]
36	*Equus caballus*	Horse	37.5	466	25.6	119	7.0	820	[94]
37	*Ursus arctos*	Brown bear	37.5	343	5.5	19	9.7	696	[95]
38	*Lontra canadensis*	North American river otter	38	486	27.2	132	7.5	839	[96]
39	*Octodon degus*	Degu	38.5	508	13.8	70	7.1	912	[97]
40	*Bos taurus*	Domestic cow	38.5	425	17.2	73	7.2	759	[98]
41	*Canis lupus familiaris*	Domestic dog	38.5	462	27.5	127	6.95	897	[94]
42	*Capra hircus*	Domestic goat	39	346	18.5	64	7.2	701	[94]
43	*Capricornis sumatraensis*	Sumatran serow	38.5	112	6.25	7	6.7	466	[99]
44	*Oryctolagus cuniculus*	European rabbit	39	524	21.8	114	7.0	1,022	[94]
45	*Sus scrofa*	Domestic pig	39	366	16.9	62	9.1	724	[94]
46	*Cavia porcellus*	Guinea pig	39.5	338	16.0	54	9.0	688	[100]
47	*Ochotona princeps*	American pika	40	309	24.0	74	6.3	663	[101]

**Fig 1:**
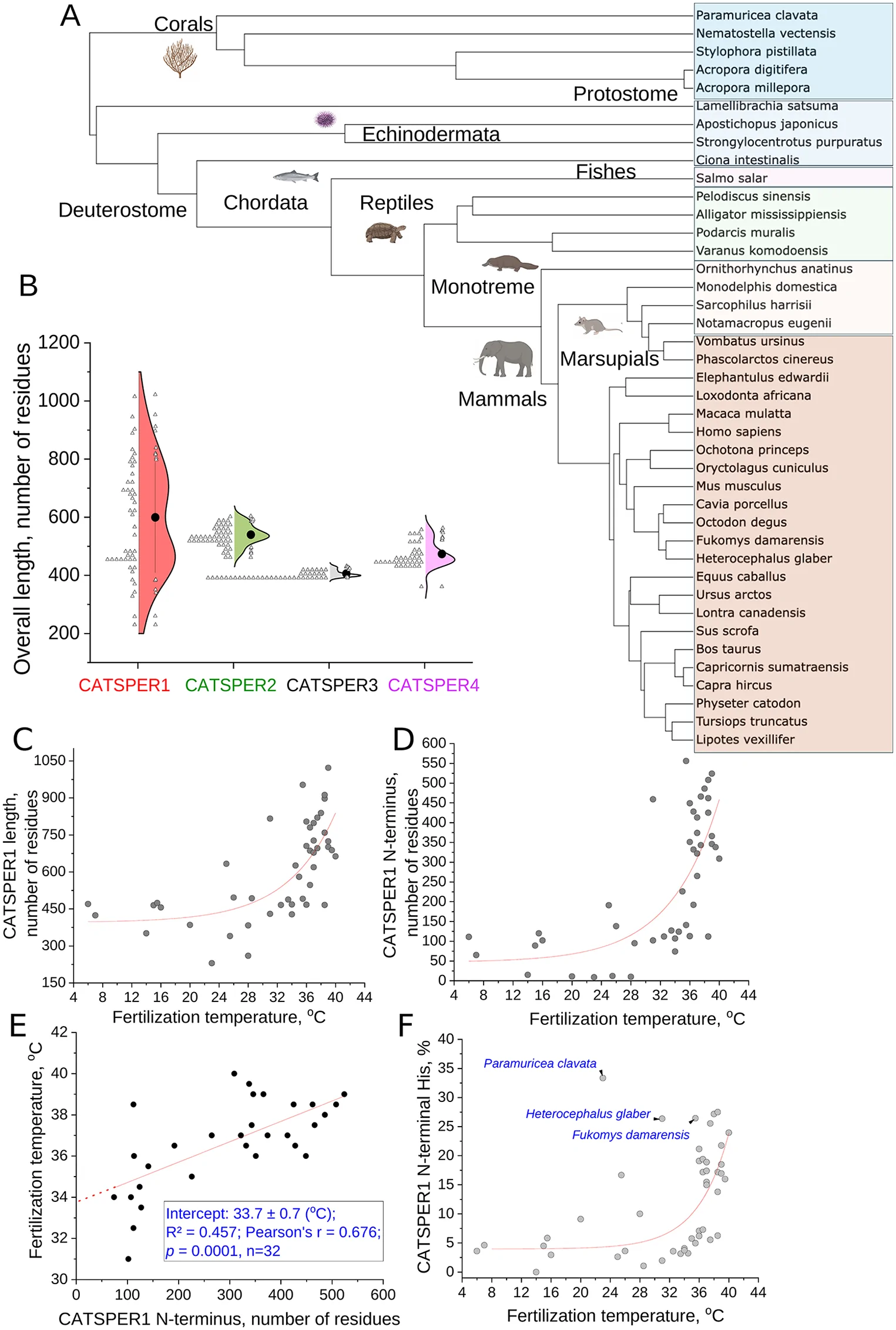
Comparative genomic analyses reveal temperature-dependent divergence of the CatSper1 N-terminus across CatSper-bearing species. (Panel **A)** Phylogenetic tree of representative species that retain CatSper channels. Created in BioRender. Lishko, P. (2026) https://BioRender.com/a1bmcre. (Panel **B)** Comparison of the total amino acid length of the four CatSper pore-forming subunits across the species shown in (Panel A), highlighting pronounced divergence of CatSper1 relative to CatSper2–4. (Panel **C)** Total CatSper1 length plotted against fertilization temperature for the species shown in (A), revealing an exponential relationship. (Panel **D)** Length of the CatSper1 NTD plotted as a function of fertilization temperature for the same species, demonstrating a strong positive correlation. (Panel **E)** Linear regression analysis using 32 (n) internally fertilizing species with fertilization temperatures from ~30 °C and higher. Pearson correlation analysis shows significant positive correlation between the variables (Pearson’s r = 0.675, *n* = 32, *P* < 0.0001, R^2^ = 0.456. Dotted line shows x-intercept at 33.6 ˚C. (Panel **F)** The percentage of histidine residues within the CatSper1 N-terminal domain shows a strong positive correlation with fertilization temperature across CatSper-bearing species shown in Fig 1A. Outliers (blue) correspond to mole rat species, which inhabit subterranean, hypercapnic environments that result in chronically acidic systemic conditions due to elevated carbon dioxide levels. The unusually high apparent percentage of N-terminal histidines in corals *(Paramuricea clavata)* reflects the exceptionally short length of their CatSper1 N-terminal domain (9 residues). Underlying data can be found in S1 Data file.

Because fertilization temperature spans a wide evolutionary range and NTD length varies substantially across taxa, we used Bayesian regression to quantify uncertainty and assess the robustness of the temperature–NTD length association (S2 Fig). This analysis supported a strong positive posterior association between fertilization temperature and CatSper1 NTD length, with the posterior distribution of the slope parameter overwhelmingly shifted toward positive values (P(β > 0 | data) > 0.99). Cold-fertilizing species, such as marine invertebrates and fishes (6–23 °C) exhibited short N termini (≈ 9–120 aa), whereas warm-fertilizing species (35–40 °C) displayed substantially expanded NTDs (≈ 200–550 aa), with intermediate temperatures showing intermediate NTD lengths. This relationship was consistent across phylogenetic distances (Figs 1D, S2A and Table 1).

To further evaluate the relationship between fertilization temperature and CatSper NTD length, we performed linear regression analysis on 34 internally fertilizing species with fertilization temperatures of approximately 30 °C or higher (Table 1). Pearson correlation analysis revealed a moderate positive correlation between the two variables (Pearson’s r = 0.510, *n* = 34 species, *P* = 0.002; R^2^ = 0.260; S2B Fig and Table 1). Because two mole rat species (*Heterocephalus glaber* and *Fukomys damarensis*) exhibited exceptionally low fertilization temperatures relative to the remaining mammals and represented clear biological outliers, we repeated the analysis after excluding these species. This analysis revealed a stronger positive correlation (r = 0.675, *n* = 32, *P* < 0.0001; R² = 0.456; Fig 1E). Interestingly, the estimated x-intercept of the regression was 33.6 ˚C, closely matching the reported threshold temperature for CatSper activation [17].

CatSper1 NTD’s extraordinary variability suggests it may serve as an evolutionary tuning module, potentially enabling species-specific modulation of CatSper activation in response to reproductive thermal environments. The presence and functional importance of the CatSper1 NTD, that features rich histidine content, has puzzled researchers since its original discovery [1,9]. While it was initially proposed to play a key role in CatSper pH gating, no experimental evidence has yet confirmed this function. The NTD of CatSper1 represents an extreme example of histidine enrichment, exhibiting the highest histidine density reported among pH-sensitive ion channels (Table 2). In mouse CatSper1, histidine accounts for 9.2% of the full-length protein, whereas the N-terminal domain alone contains ~17.2% histidine. This level of enrichment places the CatSper1 N-terminus within the range of classical histidine-rich proteins, such as histidine-rich calcium-binding protein (HRC) and histidine-rich glycoprotein (HRG), which contain ~11%–12.7% histidine (Table 2). This histidine enrichment of N-terminal region was absent from other pore forming subunits of CatSpermasome, such as CatSper2–4 across the entire evolutionary tree (Figs 1B and S3A).

**Table 2:** Histidine (His, H) abundance in known histidine-rich proteins. Histidine-rich proteins are defined by an unusually high abundance of histidine residues. In the average proteome, histidine constitutes approximately 2%–3% of amino acids; by contrast, histidine-rich proteins typically contain ≥5%–10% histidine, with some domains exhibiting substantially higher levels. Among all pH-sensitive ion channels (blue), only CatSper1 and CatSper N-terminus (CatSper1-NT; red) shows high His content, on par with other known His-rich proteins.

Protein name	Protein ID	Length (residues)	His (residues)	His content, %	Species
Histidine-rich calcium-binding protein (HRC)	P23327	699	89	12.7	*H. sapiens*
Histidine-rich glycoprotein (HRG)	Q9ESB	525	58	11	*M. musculus*
Histatin (HTN1)	P15515	57	7	12.3	*H. sapiens*
CATSPER1	Q91ZR5	686	63	9.2	*M. musculus*
CATSPER1-NT	Q91ZR5	351	58	17.2	*M. musculus*
TRPA1	Q8BLA8	1,125	46	4.09	*M. musculus*
TRPV1	Q704Y3	839	13	1.55	*M. musculus*
ASIC1	Q6NXK8	526	14	2.66	*M. musculus*
KCNK9	Q3LS21	402	10	2.49	*M. musculus*

In many histidine-rich proteins, clusters of His residues confer sensitivity to pH, metal ions, and other environmental cues, prompting the hypothesis that CatSper1’s N-terminal His clusters contribute to temperature-dependent gating. To test this, we first quantified the total histidine content and found a strong positive correlation with species-specific fertilization temperatures (Fig 1F), whereas the histidine content of CatSper2–4 showed no correlation (S3A Fig). Interestingly, an inverse relationship was observed between fertilization temperature and the predicted isoelectric point of the CatSper1 N-terminus (S3B Fig), consistent with increasing histidine enrichment conferring enhanced pH sensitivity at higher temperatures.

Secondary structure analysis of the CatSper1 NTD indicates that it possesses hallmark features of an intrinsically disordered region (IDR). These are protein regions that are defined by sequence-specific conformational heterogeneity. Consistent with this prediction, recent high-resolution cryo-EM and in situ 3D cryo-ET structures of the murine CatSper complex [21,31], obtained at 25 °C, resolved the architecture of most CatSpermasome subunits but failed to visualize the CatSper1 NTD. The absence of defined density for this region further supports the notion that the NTD is intrinsically disordered.

IDRs are defined by “molecular grammars”. This refers to non-random amino acid compositions (the alphabet) and non-random patterning of distinct residue types with respect to one another (the syntax) [42,43]. Given the disordered nature of CatSper1 NTD, we performed an unbiased analysis of molecular grammars using the NARDINI+ algorithm. This algorithm takes an IDR sequence as input and quantifies 90 different z-scores for different compositional and patterning features. Specifically, NARDINI+ identifies non-random grammar features from z-scores calculated by comparing compositional features to the human IDRome and patterning features to 10^5^ composition matched scrambles. When applied to the CatSper1 NTD sequences across evolution, the NARDINI+ analysis helped in identifying features of the IDR grammars that differed among warm-, intermediate, and cold-fertilizing species. We find that 24 grammar features have distinct z-score distributions between fertilization temperatures as assessed via the Kolmogorov–Smirnov test (Fig 2A–2C).

**Fig 2:**
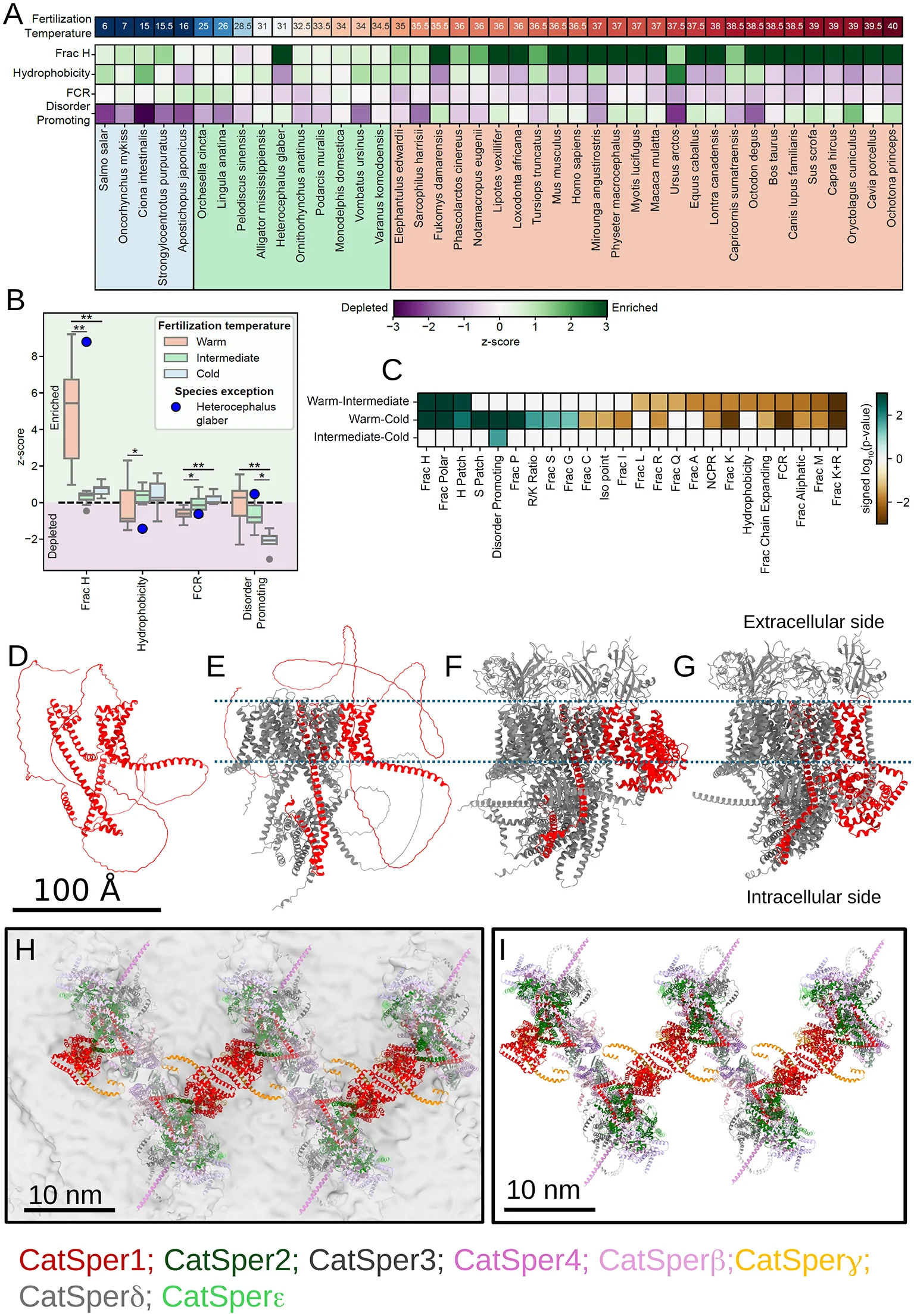
CatSper1 histidine content positively correlates with fertilization temperature. (Panel **A)** Heatmap of NARDINI+ z-scores for CatSper1 N-terminal domains per species for grammar features highlighted in (Panel **B)**. Green denotes features enriched and purple denotes features depleted relative to the human IDRome. Species were split into three fertilization temperature classes: cold (6–23 °C), intermediate (25–34.5 °C), and warm (35–40 °C). Colored boxes around the species names denote cold- (blue), intermediate- (green), and warm- (red) fertilizing species. (Panel B) Box-plots of z-scores extracted from NARDINI+ for grammar features within CatSper1 N-terminal domain that show significant differences in z-score distributions between warm-, intermediate-, and cold-fertilizing species (*: *p*-value < 0.05 and ≥ 0.001, **: *p*-value < 0.001). Z-scores greater than zero are enriched compared to the human IDRome, whereas z-scores less than zero are depleted compared to the human IDRome. The naked mole rat (*Heterocephalus glaber*) is shown as an outlier intermediate-fertilizing species by blue circles. (Panel **C)** Comparison of NARDINI+ z-score distributions of CatSper1 NTDs for grammar features between fertilization temperatures. The heatmap denotes the signed log_10_(*p*-value) as assessed via the Kolmogorov-Smirnov test. Only grammar features with *p*-values < 0.05 are shown. Here, positive values (blue-green) denote that the temperature class listed first had a mean z-score greater than or equal to the temperature class listed second. Negative values (brown) denote that the temperature class listed first had a mean z-score less than the temperature class listed second. (Panels D-G) AlphaFold3 modeling of the murine CatSper1 N-terminal region. (Panel **D)** The AlphaFold3 predicted structure of a lone murine CatSper1, shown in red. NTD is not predicted to fold into any secondary structures. (Panel **E)** AlphaFold3 predicted structure of the core subunits CatSper1-4, with CatSper1 shown in red. The NTD is still unstructured. (Panel **F)** The AlphaFold3 predicted structure of the transmembrane and cytoplasmic parts of the CatSper complex when including intracellular subunits and the transmembrane helices of the extracellular subunits. The NTD is predicted to sit in a region occupied by the cell membrane. (Panel **G)** The AlphaFold3 predicted structure of the transmembrane and cytoplasmic parts of the CatSper complex as in (Panel E), but with 50 oleic acid molecules added to the prediction. The free oleic acid clusters around the transmembrane domain, causing the NTD to adopt an intracellular position. Each structure is shown with a 100 Å scale bar. Dotted lines show plasma membrane bilayer. (Panel **H)** Integrative structural model of multiple adjacent murine CatSpermasomes fitted into the cryo-ET density of murine CatSpermasome from [21]. (Panel **I)** Same as (H) in the absence of Cryo-ET density. Single-particle cryo-EM structures and AlphaFold3-predicted models were combined to visualize the arrangement of pore-forming and auxiliary subunits within the array. Subunits are color-coded as indicated: CatSper1 (red), CatSper2 (green), CatSper3 (gray), CatSper4 (purple), CatSperβ (light pink), CatSperγ (yellow), CatSperδ (dark gray), and CatSperε (light green). Underlying data can be found in S1 Data file.

The z-scores for the fraction of histidine residues were significantly different and more enriched for warm-fertilizing species compared to intermediate- and cold-fertilizing species (Fig 2A–2C). We find that intermediate-fertilizing species are generally enriched in hydrophobic residues, whereas warm-fertilizing species are generally depleted in residues deemed to be hydrophobic (Fig 2A–2C). This result suggests that species that fertilize at intermediate temperatures may exchange histidine residues for hydrophobic residues as the drivers of temperature-dependent gating. Specifically, the strength of hydrophobic interactions can increase with increasing temperature [44]. Lastly, cold-fertilizing species show unique grammar features including an increase in the fraction of charged residues (FCR) and a decrease in disorder promoting residues compared to warm- and intermediate-fertilizing species.

In addition, the CatSper1 NTD displays a high predicted droplet-promoting propensity (pDP score approaching 1.0), suggesting a strong likelihood of liquid–liquid phase separation behavior. High pDP values (~1.0) are characteristic of proteins capable of droplet formation and self-assembly into higher-order condensates, processes that are often sensitive to environmental factors such as temperature and ionic strength. Thus, phase separation mediated by the CatSper1 IDR may contribute to the distinctive regulatory properties of CatSper1 (S4 Fig).

### Comparative genomics combined with AlphaFold3 modeling further supported a relationship between CatSper1 surface-exposed histidine residues and fertilization temperature

Next, to determine whether the CatSper1 IDR can adopt a stable three-dimensional structure under physiological conditions, we integrated AlphaFold3 predictions with available structural data. Specifically, we modeled the murine CatSper1 NTD in isolation, in the context of neighboring CatSpermasome components, and within a lipid membrane environment to assess whether molecular context promotes structural stabilization (Fig 2D–2I).

As shown in Fig 2D, initial modeling of CatSper1 NTD in isolation did not predict the formation of defined secondary structural elements, consistent with its intrinsically disordered nature. However, when the transmembrane and cytoplasmic components of the CatSper complex were included, comprising the intracellular subunits and the transmembrane helices of extracellular subunits, the NTD was positioned within a region corresponding to the plasma membrane interface (Fig 2E–2F). Upon further incorporation of free oleic acid molecules clustering around the transmembrane domain to better approximate a lipid environment, the NTD adopted an intracellular orientation (Fig 2G). This positioning is consistent with its proposed localization between adjacent CatSpermasomes [32,45,46] and enabled quantitative assessment of the density of surface-exposed histidine residues within the NTD. The calculated histidine surface density was then plotted against species-specific fertilization temperatures, revealing a strong positive correlation across mammals (S3C Fig).

Two exceptions- *Heterocephalus glaber* and *Fukomys damarensis*- possess extremely high N-terminal His content (~26%) yet intermediate core body temperatures (30–36 °C; Figs 1E–1F, 2A–2C, S2B, S3B–S3C, S5 and Table 1). These subterranean mole rats inhabit hypoxic, CO_2_-rich burrows, likely imposing unique selective pressures on sperm physiology. Such an environment may have contributed to lineage-specific adaptations in CatSper channel function and regulation, setting these species apart from other mammals in the evolutionary trajectory of sperm motility control.

To identify a potential interaction module between CatSper1 N-termini, we fitted the high-resolution cryo-EM structure of the CatSpermasome [31] into the density maps of CatSpermasome arrays previously resolved by cryo-electron tomography (cryo-ET [21]. The predicted intracellular and transmembrane architecture of the CatSper complex was then integrated into the resulting 3D reconstruction (Figs 2H–2I and 3).

**Fig 3:**
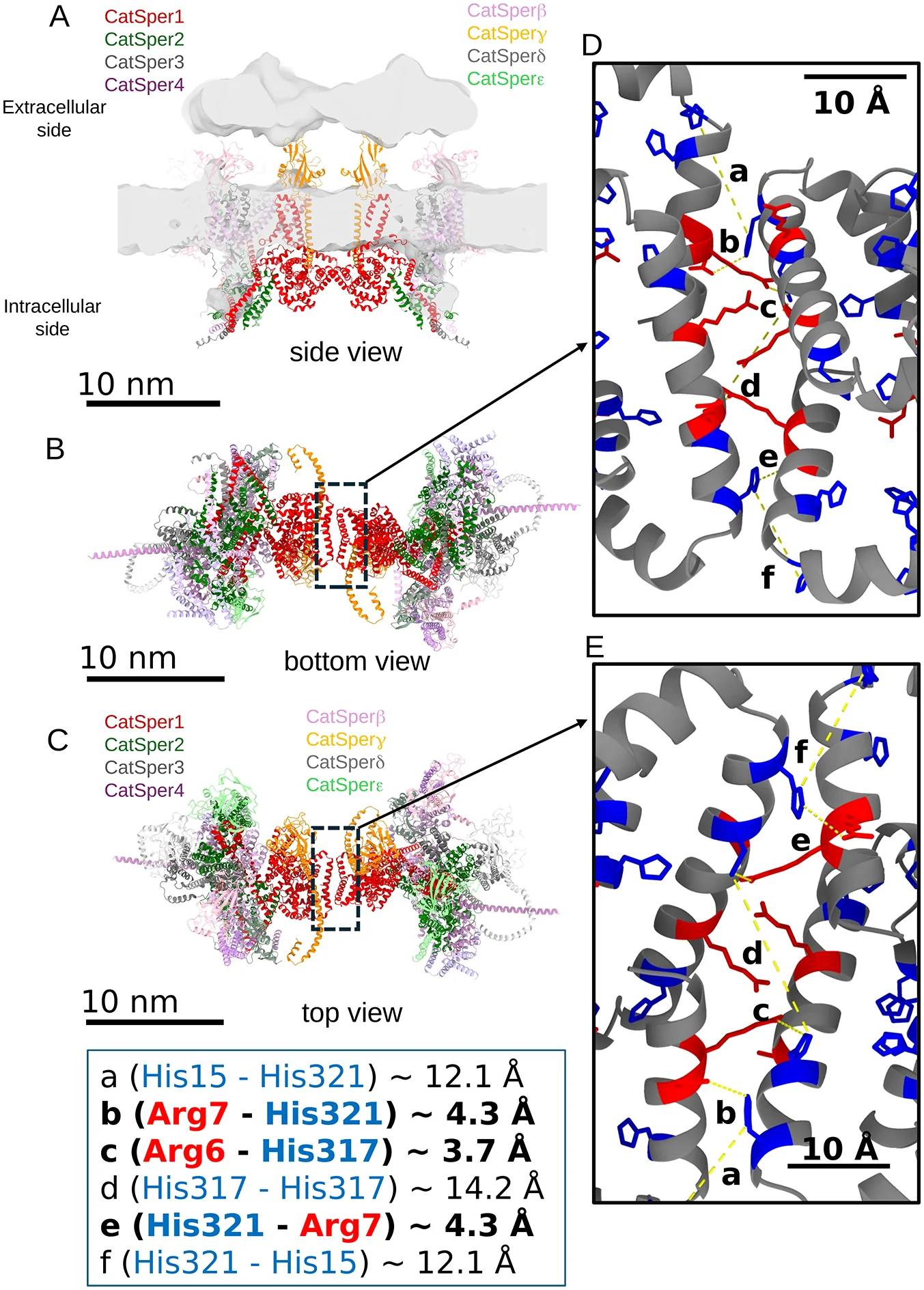
Structural modeling identifies a putative CatSper1 N-terminal interaction interface between neighboring CatSpermasomes. (Panels **A–C**) Integrative model of two adjacent CatSpermasomes viewed from the side (Panel A), bottom (Panel B), and top (Panel C). Models were generated by fitting the high-resolution single-particle cryo-EM structure of the CatSper complex from [31] into the in situ cryo-electron tomography (cryo-ET) density of CatSper arrays from [21], followed by incorporation of AlphaFold3-predicted transmembrane and intracellular regions. Pore-forming subunits are color-coded: CatSper1 (red), CatSper2 (green), CatSper3 (gray), and CatSper4 (purple). Dashed boxes indicate the region of closest approach between adjacent CatSpermasomes. Only first 290 amino acids of CatSper auxiliary subunits β, δ, ε, and γ are shown. (Panel **D)** Magnified view of the boxed region in (Panel B), highlighting paired α-helical elements from the CatSper1 NTDs positioned at the inter-complex interface. Histidine side chains are shown in blue, arginine side chains are shown in red. Dashed yellow lines indicate distances between conserved His-His residues and His-Arg residues across neighboring complexes: **a**, His15–His321 (~12.1 Å); **b**, Arg7–His321 (~4.3 Å); **c**, Arg6–His317 (~3.7 Å); **d**, His317–His317 (~14.2 Å); **e**, His321–Arg7 (~4.3 Å); and **f**, His321–His15 (~12.1 Å). (Panel **E)** Magnified view of the boxed region in (C), illustrating the spatial distribution of surface-exposed histidine residues along the interacting helices. Scale bars, 10 nm (Panels A–C) and 1 nm (Panels D, E).

This composite model revealed a previously unrecognized putative interaction interface formed by the proximal regions of CatSper1 N-terminal domains, spanning residues 6–321 in murine CatSper1. These segments are positioned at the junction between adjacent CatSpermasomes within the longitudinal arrays, suggesting a role in inter-complex coupling. AlphaFold3 predicts that this interaction module is composed of paired α-helical elements contributed by opposing CatSper1 N-termini. Each helix contains three conserved histidine residues (H15, H317, and H321; Figs 3 and S5). The spatial arrangement of these residues defines a potential interaction network, including contacts between H15 from one CatSpermasome and H321 from an adjacent complex, as well as reciprocal interactions between opposing H317 residues (Figs 3A–3C and S5).

Emerging evidence indicates that histidine residues within IDRs can mediate context-dependent stabilization [47–49]. In addition to π–π or His–His interactions, neutral histidines at pH values above ~6.8 can participate in strong cation–π interactions with positively charged residues such as arginine and lysine, promoting local structural ordering. Notably, two arginine residues (Arg6 and Arg7) are located within the predicted murine CatSper1 NTD interface. The modeled distances between these arginines and neighboring histidines (3.7–4.3 Å) fall within the range compatible with stabilizing cation–π interactions (3–6 Å). Such proximity suggests a potential mechanism for pH-dependent inter-CatSpermasome coupling mediated by Arg–His interactions (Figs 3A–3C and S5–S6).Our modeling is based on the available cryo-ET [21] map (Fig 2G) obtained at room temperature (25 °C), which is below the activation threshold of murine CatSper [17]. This likely explains the relatively long distances observed between putative interacting histidines (H317–H317 and H15–H321), which range from ~11.8 to 14.2 Å—too large to support direct π–π or hydrogen-bond interactions. We therefore propose that elevated temperature promotes histidine deprotonation, reducing electrostatic repulsion and enabling closer association of CatSper1 NTDs. Consistent with this idea, phylogenetic analysis reveals that H317 (in murine CatSper1) is the most conserved histidine residue within this interface, with notable divergence in cold-blooded internal fertilizers such as reptiles, supporting a role in temperature-dependent regulation (Figs 3 and S5). Together, these findings identify a histidine-arginine-based interface that may mediate pH- and temperature-dependent coupling between CatSpermasomes.

Histidine-rich proteins are known to serve diverse regulatory functions, including pH buffering, metal ion coordination, and modulation of protein stability. Importantly, the pKa of histidine (~6.3) [50] allows it to readily switch between charged and neutral states within a narrow physiological pH range (6.5–7.5), which coincides with the conditions that regulate CatSper activity.

In temperature-sensitive proteins, increased temperature can shift local pKa values, weakening hydrogen bonding networks and promoting histidine deprotonation. Conversely, under acidic conditions, histidine protonation leads to charge accumulation that can destabilize protein structure by introducing electrostatic repulsion and disrupting hydrophobic packing. In fact, as we have shown recently, cytoplasmic acidification to 6.0 profoundly attenuated CatSper’s temperature gating [17], which further supports CatSper N-terminal involvement in this process.

### Experimental evidence indicates that removal of CatSper1 N-terminus results in the loss of temperature-dependent gating

In our recent work, we demonstrated that murine spermatozoa subjected to prolonged capacitation (>60 min) progressively lose temperature-dependent gating of CatSper [17]. This loss is accompanied by a time-dependent decline in CatSper-mediated currents for both divalent (Ba^2+^; Fig 4A–4C) and monovalent (Cs^+^) [17]. Using direct sperm patch-clamp recordings, we observed a progressive reduction in temperature-dependent divalent conductance through CatSper and significant decrease of its temperature sensitivity after prolonged sperm capacitation (Fig 4A–4C).

**Fig 4:**
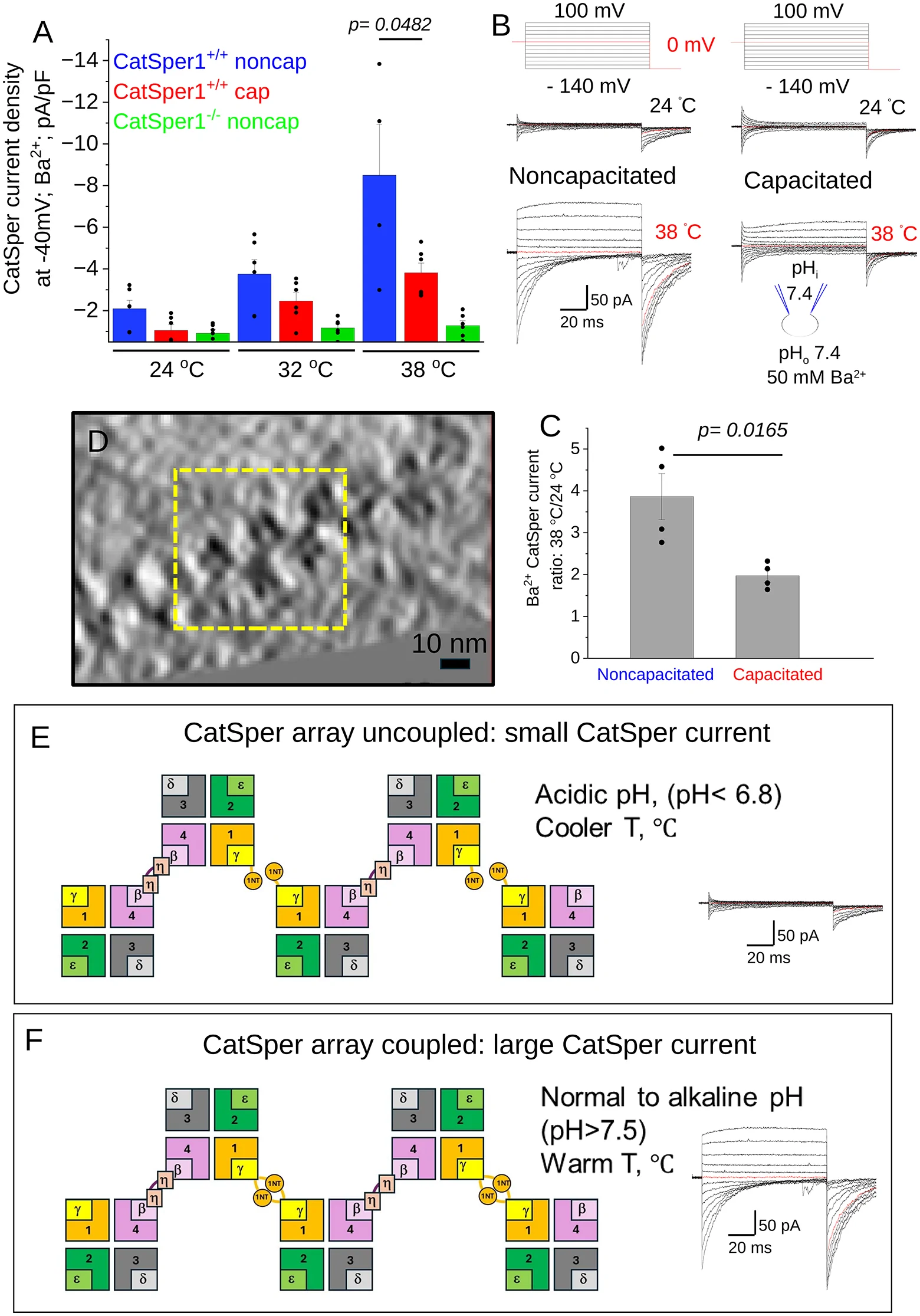
Sperm capacitation leads to removal of temperature sensitivity. (Panel **A)** Decrease in current density of Ba^2+^ currents via CatSper with sperm capacitation (red; cap) after response to elevated temperatures. CatSper1^−/−^ sperm (green, noncap) were used as negative control. Noncapacitated sperm (noncap) data are shown in blue. (Panel **B)** Representative divalent Ba^2+^ currents (*I*_*Ba*_^*2+*^) recorded from wild type noncapacitated mouse spermatozoa (left) and from wild type capacitated sperm cell (right) in response to indicated voltage steps under two different temperatures: 24 °C and 38 °C. (Panel **C)** Exposure to 38 °C increases *I*_*Ba*_^*2+*^ stronger in noncapacitated cells. Data are mean values ± S.E.M., n corresponds to the number of cells used; *n* = 3-6; total capacitation time more than 60–90 min. (Panel **D)** Representative cryo-electron tomography (cryo-ET) slice of the human CatSper channel array in the sperm flagellum from [21], viewed parallel to the principal piece axis. The dashed yellow box highlights a repeating CatSpermasome unit within the longitudinal zigzag array. (Panels **E, F**) Model for pH- and temperature-dependent coupling of CatSpermasomes mediated by the CatSper1 N-terminus. (Panel E) Schematic representation of CatSpermasome organization within the zigzag array under acidic intracellular pH and/or cooler temperature conditions. Under these conditions, protonation of histidine residues within the CatSper1 NTDs (1NT) is proposed to weaken 1NT interactions and reduce cooperative channel coupling: array uncoupled. Right insert: representative small CatSper current is shown. (Panel **F)** Corresponding schematic demonstrates array coupling under alkaline intracellular pH and warm temperature conditions. Deprotonation of histidine residues will ensure histidine-arginine coupling, stabilizing interactions between neighboring CatSpermasomes, and promoting cooperative coupling along the array. Representative enlarged CatSper current is shown on the right. Underlying data can be found in S1 Data file.

To independently validate these findings, we performed calcium imaging experiments to assess CatSper activity in response to its threshold activation in noncapacitated versus capacitated sperm. In agreement with electrophysiological recordings, sperm subjected to prolonged capacitation (80–110 min) failed to exhibit temperature-dependent Ca^2+^ influx. CatSper1-deficient sperm were used as controls to exclude non-CatSper-mediated effects. As shown in S7A–S7B Fig, capacitation completely abolished temperature-dependent activation of murine CatSper. It must be noted that the optimal duration of capacitation differs among mammalian species and that mouse sperm undergo capacitation over a substantially shorter time scale than sperm from larger mammals, such as bovine or human sperm [8,51–53].

Prior studies from Chung and colleagues and Ded and colleagues [14,39] convincingly demonstrated that these capacitating conditions induce selective proteolytic removal of at least ~150 amino acids, (~20 kDa) from CatSper1 N-terminus [14,39], temporally coinciding with the loss of temperature gating observed in our experiments (Figs 4A–4C and S7A–S7B). The cleaved region contains the majority of the histidine-rich N-terminal segment, as well as N-terminal arginines, thereby removing a substantial fraction of the residues that is proposed to contribute to temperature-sensitive interactions. Together, these findings support the conclusion that temperature gating is critical for maintaining CatSper channels organized within CatSpermasomes in a highly ordered and synchronized array, enabling their concerted opening. Disruption of this domain uncouples channels from the ordered CatSpermasome lattice, leading to asynchronous or inefficient channel opening. Loss of concerted gating reduces effective channel conductance and limits Ca^2+^ influx, thereby impairing the robust calcium signaling required to drive hyperactivated sperm motility.

Based on these principles, we propose that the high histidine content of the CatSper1 N-terminal domain paired with adjacent positively charged residues (Arg, Lys) confers sensitivity to temperature-dependent histidine deprotonation, stabilizing inter-CatSpermasome interactions in a manner analogous to alkaline pH. In contrast, intracellular acidification would promote histidine protonation, destabilizing the N-terminal interface and inhibiting channel activity (Fig 4D–4F). This model is fully consistent with experimental observations showing that CatSper is activated by warm temperatures (>34 °C) [17] and alkaline pH [6,53], while acidic pH potently suppresses channel function.

According to this model, pH- and temperature-dependent deprotonation of surface-exposed histidine residues within the CatSper1 N-termini, including His317 and His321, stabilizes cation–π interactions with Arg6 and Arg7, and promotes coupling between adjacent CatSpermasomes, thereby enabling synchronized channel activation at elevated temperatures (Fig 4D–4F).

## Discussion

In addition to regulation by pH and temperature, CatSper activity is strongly modulated by membrane voltage, such that progressively greater depolarization is required to open the channel under acidic conditions [6,17,19,53,54]. Conversely, at warmer temperatures, CatSper opens at less depolarized potentials, whereas acidic pH completely abolishes temperature-dependent gating. This interdependence between temperature, pH, and voltage suggests a shared structural basis for polymodal regulation.

We propose that this integration is mediated by the spatial proximity of the CatSper1 N-terminal interaction module to the S4 segment of the CatSper1 voltage-sensor domain (VSD). The S4 segment of CatSper1 is the strongest voltage-sensing element among the pore-forming subunits, harboring an unusually high density of positively charged residues—four arginines (Arg; R) and two lysines (Lys; K)—that together form a classical voltage-gated motif (RxxRxxRxxR; S7C Fig and [9,31]. This charge density is predicted to confer heightened voltage sensitivity relative to CatSper2–4, whose S4 segments contain progressively fewer charged residues, with CatSper4 harboring only two Arg and one His. These structural differences point to a hierarchical organization of gating contributions, with CatSper1 acting as the dominant voltage sensor and the remaining subunits serving modulatory roles.

The close apposition of the CatSper1 VSD and NTD suggests that these elements function as a coupled gating unit, integrating voltage sensing with temperature- and pH-dependent regulation. Upon membrane depolarization, upward movement of the S4 helix is expected to mechanically displace the adjacent NTD. Based on the predicted organization of CatSpermasome arrays, neighboring CatSper channels may interact through their NTD, potentially via coiled-coil–like associations. In this configuration, depolarization-induced S4 movement would impose mechanical tension on the N-terminal interface, promoting coordinated conformational transitions and priming adjacent channels for synchronized opening.

Inter-CatSpermasome coupling interfaces have previously been identified within the canopy region and at the interface between the CatSper4 and CatSperβ subunits [45,46]. However, the interface formed by the CatSper1 NTD has remained unresolved and was largely considered an intrinsically disordered region with unknown functional properties. To our knowledge, this is the first report assigning a specific structural and functional role to the CatSper1 NTD in coordinating channel opening within longitudinal CatSper arrays.

Recent quantum chemical analyses by Calinsky and colleagues [47–49] have demonstrated that histidine residues within intrinsically disordered domains can mediate pH-dependent structural assembly through dual π–π and cation–π interactions. At physiologically relevant alkaline pH, deprotonated (neutral) histidine can form stabilizing cation–π interactions with positively charged residues such as arginine (Arg) and lysine (Lys). Notably, two arginine residues are present within the predicted interface between CatSper1 N-termini, supporting the possibility of such stabilizing interactions under alkaline conditions.

In contrast, at acidic pH, protonated histidine preferentially engages in cation–π interactions with aromatic residues (e.g., Tyr, Phe, Trp), interactions that are typically stronger than those formed by neutral histidine with cationic residues. Importantly, aromatic residues are absent from the predicted CatSper1 NTD interface, suggesting that acidification would not promote alternative stabilizing contacts.

Although the arginine residues predicted to mediate cation–π interactions within the murine CatSper1 NTD appear to be poorly conserved across warm-fertilizing species, their functional role may be substituted by lysine residues (S6 Fig). Lysines are more broadly conserved among these species and are positioned in close proximity to the predicted interface region, making them plausible alternative partners for stabilizing cation–π interactions with histidines. Nevertheless, high-resolution atomic structures of CatSper complexes from additional species will be required to directly test and validate this hypothesis.

An additional important variable that may influence histidine abundance in CatSper1 is the pH of female reproductive tract that varies during estrous and menstrual cycles. Indeed, during the fertile period, cervical mucus becomes markedly more alkaline, reaching pH values as high as ~9.0 in some species, thereby facilitating sperm transport [53,55–57]. Such physiological pH fluctuations could influence the protonation state of histidine residues and thereby modulate the function of pH-sensitive proteins.

Histidine is a particularly effective physiological buffer because its imidazole side chain has a pKa of approximately 6.0–6.5, close to physiological pH. As environmental pH decreases, histidine residues become reversibly protonated, transiently binding excess H+ and thereby increasing buffering capacity. Consequently, proteins enriched in histidine residues generally exhibit greater tolerance to transient acidification, as reversible protonation helps preserve protein structure and function over a wider pH range.

In such cases, species that inhabit chronically hypoxic and hypercapnic underground burrows, such as naked mole rat (*Heterocephalus glaber*), could present a condition that is expected to promote tissue acidification, particularly of the female reproductive tract. Combined with reduced thermogenesis and lower core body temperature reported for these species, these environmental pressures may have favored proteins with enhanced buffering capacity. We therefore suggest that the increased abundance of histidine residues in naked mole rat CatSper1 could provide greater local buffering, helping to stabilize the channel and preserve its function under conditions of fluctuating pH. At present, however, this hypothesis cannot be directly evaluated because reproductive tract pH has not been measured in naked mole rats*.*

Together, these findings identify a histidine-dependent coupling interface positioned adjacent to the dominant voltage-sensing element of the CatSpermasome, providing a structural framework for the coordination between temperature, pH, and voltage in CatSper gating. In this model, acidic pH is predicted to disrupt N-terminal coupling by protonating histidine residues within the interface, increasing electrostatic repulsion and weakening inter-channel interactions. This would reduce coupling efficiency, leading to asynchronous channel opening and diminished cumulative Ca^2+^ influx. Conversely, elevated temperature and alkaline pH would favor histidine deprotonation, stabilize N-terminal interactions, and enhance cooperative and, moreover, synchronized gating within CatSper arrays (Fig 4E – 4F). More broadly, this work establishes CatSper as a new model for how evolutionary pressures sculpt ion-channel architecture to integrate environmental and physiological signals essential for optimal sperm performance.

As we recently reported [58], the estimated unitary conductance of mammalian CatSper appears to be remarkably low. This estimate is based on the ~6,000 CatSper complexes present along the murine sperm flagellum, as determined by cryo-EM and cryo-electron tomography analyses [21,31,46,58]. Assuming this channel density, the calculated unitary conductance of an individual CatSpermasome ranges from ~121 fS to 1.2 pS, indicating that CatSper is an exceptionally low-conductance, yet highly calcium-selective, ion channel. Given such a small unitary conductance, the large macroscopic CatSper currents routinely recorded from mammalian sperm are unlikely to result from the stochastic opening of individual channels. Instead, these currents likely require the coordinated activation of thousands of CatSper complexes assembled into supramolecular arrays along the flagellum. It is therefore feasible to assume that cooperative coupling between neighboring CatSpermasomes promotes their synchronized opening, providing a mechanistic explanation for how a sperm cell can generate robust macroscopic calcium currents despite the extremely low conductance of individual CatSper channels.

The model presented here is grounded in comparative genomics, structural modeling, and integration of high-resolution cryo-EM and cryo-ET data with functional analysis of murine sperm cells. However, definitive validation of the proposed histidine-dependent coupling mechanism will require atomic resolution of CatSper conformational dynamics across defined pH, temperature, and voltage conditions. In particular, high-resolution structural snapshots captured under physiologically relevant temperatures and targeted mutagenesis of conserved histidine residues will be essential to resolve the precise molecular rearrangements underlying synchronized gating. Such experiments are beyond the scope of the present study but are a natural and necessary extension of this work, and we anticipate that future experimental structures will further refine and strengthen the mechanistic model proposed here.

## Methods

### Comparative genomics and sequence alignment

Protein sequences for CatSper subunits and related proteins across a wide range of species were retrieved using Python Scripts accessing the UniProtKB, UniParc, and NCBI APIs. Data extraction was done in June 2025. Sequences labeled as partial or containing the names of other subunits were removed. In cases where a single species had multiple isoforms, the longest sequence was selected. Suspected misannotated sequences were visually identified in an alignment and replaced using NCBI Protein BLAST searches with the corresponding mouse subunit. Species with at least 4 pore-forming subunits (Catsper1–4) conserving pore-forming subunits (Catsper1–4) were chosen for sequence alignment. An evolutionary tree was built using NCBI Common Tree [PMID: 32761142].

### Definition of the CatSper1 N-terminal region

To define the boundaries of the Catsper1 N-terminal region, all full-length Catsper1 protein sequences from the dataset were aligned using MUSCLE (v5.2) [102] with default parameters. The resulting alignment was used to identify the start of the conserved pore-forming region, defined as the first position in the alignment containing a block of at least 10 consecutive columns with greater than 80% occupancy. For each individual sequence, the non-gap characters in the alignment preceding this position were used as the N-terminal region. The N-terminal sequence spanned from the beginning of the sequence to the beginning of the short 20 amino acid (murine) linker region connecting the N-terminal domain to the S1 transmembrane domain.

### Quantification of N-Terminal histidine content

To calculate N-terminal histidine abundance, the number of histidine residues in an N-terminal sequence was normalized to the total number of amino acids in the N-terminus.

### Computation of physicochemical properties of the CatSper1 N-terminus

Theoretical isoelectric points for each N-terminal sequence were calculated using the Expasy ProtParam web server [103]. Liquid-Liquid Phase Separation (LLPS) probabilities were calculated using the FuzDrop [104] web server, using the mouse N-terminal sequence as a representative model. A pDP score greater than 0.6 was used as the threshold for identifying likely LLPS regions.

### Quantification of grammar differences between warm-, intermediate, and cold-fertilizing species

To perform an unbiased assessment of grammar differences between species that fertilize at different temperatures, we ran NARDINI+ utilizing NARDINI 2.0 (May 2026) [42,43]. NARDINI+ quantifies z-scores of 54 compositional and 36 patterning grammar features in order to determine non-random grammars within an IDR sequence. This analysis was only performed on CatSper1 NTDs of length ≥ 30 to avoid erroneous interpretations from short sequences when calculating features based on fractions of a given residue or residue type.

The 54 compositional features are: the fraction of each of the 20 amino acids (20 features); patch parameters defined as the fraction of the IDR comprised of a given residue or RG stretches (20 features); the fraction of polar (Asn, Cys, Gln, Gly, His, Ser, Thr), aliphatic (Ala, Ile, Leu, Met, Val), aromatic (Phe, Trp, Tyr), K + R (Lys, Arg), D + E (Asp, Glu), charged (FCR), chain expanding (FCE), and disorder promoting residues (8 features); the ratios of Arg to Lys (R/K Ratio) and Glu to Asp (E/D ratio) (2 features); and the net charge per residue (NCPR), isoelectric point, hydrophobicity, and polyproline II propensity (4 features) [105]. Z-scores for these features are calculated by comparing the feature value of the given CatSper1 NTD to the feature distribution from the human IDRome. The human IDRome contains 24,508 IDRs of length ≥ 30 and was extracted using MobiDB on the human proteome (Swiss-Prot database) [43,106,107].In general, for compositional features, a z-score value ≥ 1 implies that feature is non-randomly enriched and a z-score value ≤ −1 implies that feature is non-randomly depleted compared to the human IDRome.

The 36 patterning features consider the patterning between eight types of residues: pol = (S, T, N, Q, C, H), hyd = (I, L, M, V), pos = (K, R), neg = (E, D), aro = (F, W, Y), ala = (A), pro = (P), and gly = (G). To calculate non-random grammars of patterning features the sequence of each CatSper1 NTD studied was scrambled 10^5^ times. The composition matched scrambles form the prior distribution of patterning of two types of residues. For patterning features, z-scores less than zero imply the input sequence is more well-mixed than expected and z-scores greater than zero imply the input sequence is blockier than expected.

To determine if CatSper1 NTDs from warm (35–40 °C), intermediate (25–34.5 °C), and cold (6–23 °C) fertilizing species house different grammars, we compared the z-score distributions for all 90 features between warm-intermediate, warm-cold and intermediate-cold fertilizing species using the Kolmogorov-Smirnov test. We identified 24 grammar features have distinct z-score distributions between fertilization temperatures (*p*-value < 0.05). All of the significantly different features were compositional features.

### Catspermasome structural modeling and bioinformatic analysis

Structural models of the transmembrane and intracellular parts of the CatSper complex were generated using AlphaFold3 [40] In cases where model inference failed, auxiliary subunits distant from the N-terminal region were excluded to improve model inference. The last 280 residues of each of the extracellular subunit sequences, containing the transmembrane helices, were included in the AlphaFold3 prediction as separate chains. Fifty oleic acid ligands were added to the Alphafold3 prediction to mimic the membrane environment. The N-terminal domain was only predicted to fold into secondary structures when the transmembrane helices of the extracellular subunits were included in the prediction (S5 Fig). Five models were generated for each complex. For each species, the model with the highest-ranking score as calculated by Alphafold3 was used for all further analysis.

Single-particle cryo-EM maps (EMD-31076) [31] were fitted as rigid bodies into the cryo-ET structure for the in situ arrangement of the CatSper complex (EMD-24210) [21] using ChimeraX [108]. Alphafold3 predicted structures for the transmembrane and intracellular regions of the CatSper complex were fitted to these cryo-EM maps to visualize the relative orientations of complexes. A residue was defined as being on the surface if its Relative Solvent Accessibility (RSA) was greater than 25%. RSA was calculated for each residue using the formula RSA = (SASA / MaxSASA) * 100. Only residues in the N-terminal region were accounted for in the calculation. The Solvent Accessible Surface Area (SASA) was calculated for each PDB model using the Shrake-Rupley algorithm as implemented in the Bio.PDB.SASA package in Biopython (v1.85) [109]. Maximum SASA values (MaxSASA) for each amino acid were taken from the reference values published by Tien and colleagues (2013) [110].

### Bayesian regression analysis

To assess the relationship between fertilization temperature and CatSper1 N-terminal (NT) length across species, we performed a Bayesian regression analysis using data summarized in Table 1 and Fig 1d. NT length was modeled as a shifted exponential function (NT_i_) of fertilization temperature:


$$\begin{array}{c} {NT}_{i}\sim N(\alpha +\beta e^{\gamma T_{i,std.}},\sigma ) \end{array}$$

where $T_{i,std.}$ represents the standardized temperature $\frac{T_{i}-\bar{T}}{\sigma_{T}}$, *α* represents the baseline NT length, *β* represents the scaling parameter of exponential growth, *γ* represents the growth rate parameter, and *σ* represents the residual variance. Priors were chosen to be weakly informative and biologically plausible, reflecting observed NT length ranges:

*α ~ N* (100,50),

*β ~ HalfNormal* (50),

*γ ~ LogNormal* (0,1),

*σ ~ HalfNormal* (50).

Posterior distributions were estimated by Markov chain Monte Carlo sampling using the No-U-Turn Sampler implemented in PyMC [111]. Four chains were run for 3,000 iterations, each following 2,000 tuning steps with a target acceptance rate of 0.95. Inference was based focused on the posterior probability distributions of all parameters.

### Linear regression analysis

Linear regression was performed to evaluate the relationship between fertilization temperature and CatSper NTD length across internal fertilizers. Pearson's correlation coefficient (r), the coefficient of determination (R^2^), and two-tailed P values were calculated to assess the strength and significance of the association. Regression analyses were performed using Origin 9.0 Software. Outlier-excluded analyses were performed after removal of the two mole rat species (*Heterocephalus glaber and Fukomys damarensis*), which exhibit exceptionally low fertilization temperatures compared with the other internally fertilizing mammals analyzed.

### Data analysis

All the data are presented as mean values ± S.E.M., unless otherwise specified, and the number of individual cells is shown as (n). For current densities calculations, the CatSper Ba^2+^ amplitudes were normalized to capacitance (Cm, pF); during data analysis capacitance artifacts are graphically removed. Post-hoc Tukey’s test was used for comparison of mean values and statistical significance.

### Animals and ethics statement

All mouse procedures were performed according to the National Institutes of Health Guiding Principles for the care and use of laboratory animals. These procedures were reviewed and approved by the Institutional Animal Care and Use Committee of Washington University in St. Louis (St. Louis, MO, USA) (protocol # 20-0126 and protocol 22-0251). C57BL/6, C57BL/6NJ, and CatSper1 knock-out male mice (CatSper KO) (60–90 days old) were purchased from Charles River and Jackson Laboratory (JAX) and kept at a constant temperature of 22 ± 2 °C under a 12/12 h dark/light cycle with free access to food and water at the barrier animal facility of WashU School of Medicine. The animals were fed a standard chow diet. There was no difference in the sperm quality between mouse lines. CatSper1^−/−^ males were born at a mendelian ratio by breeding CatSper1^−/−^ females with CatSper1^+/−^ males. Genotypes were continuously monitored by TransnetYX (TN) and verified by in-house genotyping as reported in [17].

### Mouse sperm collection and capacitation

Sexually mature male mice (60–90 days old) were euthanized using CO_2_ followed by cervical dislocation, and sperm were isolated from the cauda epididymis. Sperm were allowed to swim-up in noncapacitating (NC) TYH media buffered with HEPES (in mM: 119.3 NaCl, 4.7 KCl, 1.71 CaCl_2_.2H_2_O, 1.2 KH_2_PO_4_, 1.2 MgSO_4_.7H_2_O, 5.56 glucose, 0.51 sodium pyruvate, 10 HEPES) at pH 7.4 for 15–20 min at 37 °C, unless specified. The motile fraction of the sample was removed from the tube. To induce capacitation, sperm were incubated in capacitating (CAP) TYH medium supplemented with 5 mg/mL BSA and 15 mM NaHCO_3_ at 37 °C for 60–90 min. NC sperm were incubated in TYH without BSA and NaHCO_3_.

### Sperm intracellular calcium concentration measurement

After sperm swim-up in NC or CAP conditions for 20 min, motile sperm were collected and incubated with 10 μM Fluo-4 AM and 0.05% Pluronic F-127 at 37 °C for 60−90 min, under NC or CAP conditions. Sperm cells were centrifuged at 300−400 *g* for 10 min and further resuspended in the corresponding media. Overall, sperm cells were capacitated for a total time of 80−110 min. Cells (5 X 10^6^/well) were seeded in a 384-well black, clear-bottom plate (Greiner Bio-One) and sperm [Ca^2+^]_i_ was recorded at 25, 30, and 38 °C using a CLARIOstar plate reader (BMG LABTECH). The instrument was set to bottom-reading mode with an excitation wavelength of 483−14 nm and emission of 530−30 nm. Acquisition parameters used were: 10 flashes per well with a cycle time of 30 s per read. Gain was adjusted to 50% on the highest signal well. The protocol was set to double orbital shaking at 300 rpm for 10 s before each measurement. Data were collected with MARS Data Analysis software. For each condition, an initial 10-minute baseline reading at 25 °C was followed by sequential 10-min recordings at 30 and 38 °C, followed by a final perfusion with 10 μM Ionomycin (Iono) at the end of every experiment. Clampfit 10 (Molecular Devices), and GraphPad were used for data analysis. To compare fluorescence across different samples and experimental conditions, Fluo-4 fluorescence (F) in response to different temperatures for each sperm was normalized to its respective F_Iono_ (F/F_iono_).

### Supporting information

S1 Fig Comparative genomic analyses of CatSpermasome transmembrane subunits. (Panel A) Comparison of the total amino acid length of eight CatSpermasome core-forming subunits across species shown on Fig 1A. (Panel B) CatSper1–4 entire length plotted against fertilization temperature for the species shown in Fig 1A. Underlying data can be found in S1 Data file.
(TIFF)

S2 Fig Bayesian regression analysis supports strong positive posterior association between fertilization temperature and CatSper1 N-terminal length. (Panel **A**) Priors were chosen to be weakly informative and biologically plausible, reflecting observed NT length ranges: *α ~ N* (100,50); *β ~ HalfNormal*(50); *γ ~ LogNormal*(0,1), *σ ~ HalfNormal*(50). Parameters were inferred using Monte Carlo sampling. Four chains were run for 3,000 iterations, each following 2,000 tuning steps with a target acceptance rate of 0.95. Trace and posterior plots show convergence of the chains and the distribution of model parameters. (Panel **B**) Linear regression analysis using 34 internally fertilizing species with fertilization temperatures from 30 °C and higher. Pearson correlation analysis demonstrated a moderate positive correlation between the two variables (Pearson’s r value = 0.510, *n* (number of species) = 34, *p* = 0.002; R² = 0.260). The analysis includes two outliers (two species of mole rats *Heterocephalus glaber* and *Fukomys damarensis*). Underlying data can be found in S1 Data file.
(TIFF)

S3 Fig Correlation between CatSper1 N-terminal histidine content, pI and fertilization temperatures. (Panel **A**) The percentage of histidine residues within entire CatSper1–4 subunits plotted against fertilization temperature. Only CatSper1 shows positive correlation. (Panel **B**) Theoretical isoelectric point (pI) calculated for N-terminus domain of CatSper1 plotted against fertilization temperature for mammalian species shown in Fig 1a. (Panel **C**) Surface His density correlates with fertilization temperatures. Underlying data can be found in S1 Data file.
(TIFF)

S4 Fig The CatSper1 N-terminal domain uniquely exhibits high predicted liquid-liquid phase separation propensity. Predicted liquid–liquid phase separation (LLPS) propensity of CatSper pore-forming subunits. pDP scores calculated using FuzDrop are plotted as a function of residue position for CatSper1, CatSper2, CatSper3, and CatSper4. Domain boundaries are indicated schematically above each plot: N-terminal domain (NTD, peach), transmembrane domain (TMD, gray), and C-terminal domain (CTD, peach). The red dashed line denotes the pDP threshold of 0.6, above which regions are predicted to have a high propensity for droplet formation.CatSper1 displays an extended N-terminal region with consistently high pDP scores exceeding the LLPS threshold, whereas CatSper2–4 lack comparable LLPS-prone regions in their N-terminal domains and instead show elevated pDP scores primarily within C-terminal regions. These results indicate that a predicted intrinsically droplet-forming region is a unique feature of the CatSper1 N-terminus among the pore-forming CatSper subunits. Underlying data can be found in S1 Data file.
(TIFF)

S5 Fig Sequence alignment of CatSper1 NTDs between residues 300 and 330 of murine CatSper1 across species. Conserved His residues indicated by arrowheads.
(TIFF)

S6 Fig Sequence alignment of CatSper1 NTDs withing first 10 resides across 47 species. (TIFF)

S7 Fig Sperm capacitation leads to removal of temperature sensitivity as shown by calcium imaging. (Panel **A**) Average traces of normalized Fluo4-AM fluorescence in wild-type noncapacitated mouse spermatozoa (blue) and from capacitated sperm (red; total capacitation time 80–110 min) in response to increasing temperatures: 25 °C, 30 °C, and 38 °C. (Panel **B**) Average traces of normalized Fluo4-AM fluorescence in noncapacitated wild-type (blue, same data as above in (Panel A)) and CatSper1^−/−^ (green) mouse spermatozoa in response to increasing temperatures: 25 °C, 30 °C, and 38 °C. Each trace was normalized to its respective ionomycin (Ionomycin, 10 μM) response. Data are presented as mean and standard deviation (*n* = 3–4 biological replicates). *P < 0.050, **P < 0.010, ****P < 0.00010, ns = non-significant, by unpaired *t* test. (Panel **C**) Hierarchical organiza*t*ion of CatSper voltage-gated module located in S4 regions. Sequence alignment between S4 regions of murine CatSper1–4. An unusually high density of positively charged residues, four R and two K, is located in the S4 region of CatSper1, which is predicted to enable enhanced voltage sensitivity. CatSper2–4, however, contain progressively fewer charged residues, with CatSper4 harboring only two R. Conserved positively charged residues are indicated by green triangles. Blue triangles indicate additional residues unique to CatSper1. Underlying data can be found in S1 Data file.
(TIFF)

S1 Data Source Data used to build Figures. (XLSX)

## Abbreviations

aa: amino acid
CatSper: cation channel of sperm
CIMED: Center for the Investigation of Membrane Excitability Diseases
cryo-ET: cryo-electron tomography
FCE: chain expanding
FCR: fraction of charged residues
HRC: Histidine-rich calcium-binding protein
HRG: histidine-rich glycoprotein
IDR: intrinsically disordered region
LLPS: liquid–liquid phase separation
NC: noncapacitating
NCPR: net charge per residue
NTD: N-terminal domain
RSA: Relative Solvent Accessibility
SASA: Solvent Accessible Surface Area
VSD: voltage-sensor domain.
