# Supplementary material for: A histidine-mediated supramolecular mechanism links temperature and pH activation of the sperm channel CatSper
Source: PLoS Biol. 2026 Oct 9;24(10):e3003776. doi: 10.1371/journal.pbio.3003776 (PMC13674862; doi:10.1371/journal.pbio.3003776)
Supplement: Supplementary file 1 [file pbio.3003776.s012.pdf]

Dear *PLOS Biology* Editors,

We would like to thank all Reviewers and Editors for their constructive and helpful feedback, as well as for their time and efforts. We also appreciate the careful editorial handling of our manuscript and the opportunity to revise it. Overall, we were encouraged by the comments and believe that the vast majority of the concerns raised across all reviews have been addressed through the revisions, addition of the power analysis, new data and clarifications presented in the revised manuscript. Many of the concerns appear to stem from misunderstandings of the experimental methodology or from issues of presentation and framing, both of which we have addressed in the revised manuscript. We believe that nearly all concerns have been addressed through clarification of the experimental conditions and methodology, revision of the language and interpretation, and a clearer distinction between experimentally supported findings and mechanistic models.

One example concerns the capacitation conditions used in our calcium imaging experiments. All experiments were performed under matched capacitating conditions, including >60 minutes of capacitation sufficient for CatSper1 N-terminal processing for murine CatSper, consistent with all prior literature. We have clarified the Methods and Results sections to make this clearer. We also clarified that the current manuscript investigates potential structural and evolutionary mechanisms underlying CatSper temperature sensitivity, rather than re-establishing temperature gating itself, which was experimentally demonstrated in our previous peer-reviewed work.

The only point that we believe cannot be fully addressed at present is the request for direct structural visualization of open-state CatSper conformational transitions at atomic resolution. Given that CatSper supramolecular assemblies can presently only be studied in their native sperm environment, such experiments would require a level of in situ cryo-ET resolution that is currently beyond what is technically achievable. We have, however, explicitly discussed these limitations in the revised manuscript.

The revised manuscript has been substantially strengthened through additional analyses of CatSper IDR components performed by Drs. Kiesten Ruff and Rohit Pappu, who have been added as co-authors, the inclusion of new data and figures, and extensive revisions to the text. Together, these additions further strengthen the conclusions of the study.

Below is our point-by-point response (in italics). Please note that the figure order has been updated to reflect the changes requested. Major revisions are shown using Grey Highligths in the marked-up version of the manuscript.

REVIEWER COMMENTS

Reviewer #1: The paper by Zhao et al is a fascinating examination of the evolutionary mechanism for CatSper channel complex sensitivity to pH and temperature, enabling sperm capacitation when the sperm encounter the alkaline pH and body temperature of the vaginal fornix after intercourse. CatSper arrays, containing ~ 15 distinct subunits, are probably the most complex ion channels in nature, reflecting substantial refinement and strong evolutionary pressure. Part of its unusual nature is the length of its N terminus in higher animals, which is enriched with histidine residues (17% in mouse CatSper1 NTD, similar to other histidine rich proteins that sense pH). The N-terminal domain (NTD) of CatSper1 varies significantly across species, correlating with fertilization temperature. Histidine-arginine interactions may stabilize inter-CatSper complexes via cation-π interactions, sensitive to pH and temperature.

The experimental data appear strong. Electrophysiology shows reduced temperature-dependent currents after NTD degradation. Calcium imaging indicates capacitated sperm lose temperature gating. Proteolytic removal of the N terminus correlates with loss of temperature sensitivity. Histidine residues within the NTD mediate pH-dependent assembly via π-π and cation-π interactions, stabilized at alkaline pH with deprotonated histidines. The experimental, structural and AlphaFold data together support the hypothesis that elevated temperature and alkaline pH causes histidine deprotonation, stabilizing inter-complex coupling.

In my opinion, the paper requires no new experimental data. My suggestions are mainly for discussion unless the data are available in the literature.
A: Thank you for these encouraging comments! We have revised and expanded the Discussion section in accordance with your suggestions.

1. Perhaps this is not known, but it would be interesting to the measured pericervical pH during fertilization, particularly for the mole rat. I suspect the data is not known, but correlation of pH across species, like temperature, would be interesting. The pH variation may account from some of the deviations in the temperature plots of various species.
A: Very interesting suggestions! We are not aware of measurements of pericervical (or cervical luminal) pH during fertilization in mole rats. More generally, comparative in vivo data on reproductive tract pH are surprisingly sparse across mammals. We agree that reproductive tract pH is an important variable that could potentially interact with temperature during fertilization. Indeed, during the fertile period, cervical mucus becomes markedly more alkaline, reaching pH values as high as ~9.0 in some species, thereby facilitating sperm transport and function[1-4] (Eggert-Kruse et al., 1993; Maas et al., 1977; Lishko et al., Annu. Rev. Physiol). Such physiological pH fluctuations could influence the protonation state of histidine residues and thereby modulate the function of pH-sensitive proteins.

Histidine residues are particularly well suited to physiological pH sensing, and to act as physiological buffer because the imidazole side chain has a pKa close to physiological pH (~6.0–6.5). Consequently, changes in extracellular pH alter the protonation state of histidines, ensuring protein buffering capacity. Consequently, proteins enriched in histidine residues generally exhibit greater tolerance to transient acidification, as reversible protonation helps preserve protein structure and function over a wider pH range.

Naked mole rats inhabit chronically hypoxic and hypercapnic underground burrows, conditions that are expected to promote tissue acidification. Combined with their reduced thermogenesis and lower core body temperature, these environmental pressures may have favored the evolution of proteins with enhanced buffering capacity. We therefore speculate that the increased abundance of histidine residues in naked mole rat CatSper1 could provide greater local buffering, helping to stabilize the channel and preserve its function under conditions of fluctuating pH. At present, however, this hypothesis cannot be directly evaluated because reproductive tract pH has not been measured in naked mole rats. We have therefore added a sentence in the Discussion acknowledging reproductive tract pH as a potentially important variable that warrants future investigation.

2. The lipid droplet idea is interesting, but it would be good to reinforce with what is known about lipid droplet formation conditions and state the lipid composition of the sperm membrane containing the CatSper complex compared to other cell membranes.
A: Thank you for this suggestion! We have performed additional unbiased analysis of CatSper1 IDR molecular grammar using powerful NARDINI+ algorithm, which further supported our conclusions. We have added this data to the revised version.

Minor: Grammar
'partial degradation of N-terminal domain' should be 'partial degradation of the N-terminal domain'
', feature truncated NTD barely reaching' should be ', feature truncated NTDs barely reaching'
A: Thank you, this has been corrected.

"The modeled distances between these arginines and neighboring histidines (3.7-4.3 Å) fall within the range compatible with stabilizing cation-π interactions (3-6 A)"--- include the part in parentheses
A: Corrected.

Reviewer #2: In this interesting manuscript, the authors address a long-standing question that has intrigued researchers for over 25 years: why CatSper1, a sperm restricted ion channel protein that is fundamentally important for sperm motility and male fertility, contains so many histidine residues in its N terminus. Using evolutionary analyses, the authors make the intriguing observation that histidine content increases during metazoan evolution, correlating with increasing fertilization temperature. Using AlphaFold3 based modeling, the authors identify a potential interaction between the histidine rich N terminus and the canonical gating machinery of the channel, suggesting a mechanism for temperature sensitive channel gating mediated by the histidine rich domain. Taking advantage of previous findings showing that part of the CatSper1 N terminus is cleaved during sperm capacitation, the authors compare the temperature sensitivity of CatSper using patch clamp recordings and Ca^{2+} imaging in capacitated versus non capacitated sperm. Consistent with their hypothesis, capacitated sperm—which presumably lack part of the N terminus—exhibit reduced temperature sensitivity in channel activation.

Overall, these findings are very interesting and provide potential answers to a long standing biological problem. The authors' approach is both unique and appropriate. Importantly, this question could not have been effectively addressed using alternative strategies, given the intrinsically disordered nature of the N terminus (which precludes high resolution structural determination) and the inability to reconstitute CatSper channels in heterologous expression systems despite decades of effort. As discussed by the authors, the results presented here are also likely to stimulate future studies in the field.

Specific comments:

1. In the structural models, it would be helpful to draw a membrane and clearly indicate extracellular and intracellular spaces.

A: Thank you for this helpful suggestion. We have added membrane borders (Figure 2) and overlayed with cryo-ET membrane density, as well as indicated the extracellular and intracellular sides (Figure 3).

2. In the Introduction, the statement "The cation channel of sperm (CatSper) is a sperm specific, calcium ion channel that is essential for male fertility across the evolutionary tree" may be overstated. The cited literature (refs. 1-15) appears to demonstrate essential roles for CatSper primarily in humans and mice. Is there evidence that CatSper is essential for male fertility in additional species across the evolutionary tree?

A: Agreed. We have corrected this. CatSper has been shown to regulate sperm hyperactivation in sea urchins and has been functionally characterized in rat, mouse, human, and macaque sperm. However, genetic evidence demonstrating that CatSper is essential for male fertility is currently available only for mice and humans. We have modified the sentence that now reads: "The cation channel of sperm (CatSper) is a sperm-specific calcium ion channel that plays an important role in sperm function across the evolutionary tree and is essential for male fertility in mice and humans".

3. In Fig. 3c and d, the traces labeled "non capacitated CatSper1^{+}/$^{+}$ (WT)" in the two panels appear to be the same. If these traces are replotted, this should be clearly stated in the figure legend.

A: Thank you for pointing this out. The traces are intentionally replotted for comparison, and this is now explicitly stated in the legend to Figure 4c,d.

4. In Fig. 4b, while it is clear that capacitated sperm exhibit smaller currents across all temperatures, the temperature dependence of channel activation is less apparent. To better illustrate temperature sensitivity, it would be helpful to also plot the ratio of currents measured at 41 °C to those measured at 24 °C.

A: Thank you for this helpful suggestion. To better illustrate the temperature dependence of CatSper activation and to incorporate the requested fold-change analysis, we replaced the original recordings of monovalent Na^{+} currents with new recordings of divalent Ba^{2+} currents (Figures 4a,c). These recordings more robustly resolve the temperature-dependent activation of CatSper from

noncapacitated sperm cells, and allow comparison of the fold increase in current (38 °C/24 °C), that correlates with the Ca^{2+} imaging data presented in Figure 4. The revised data clearly demonstrate the statistically significant difference in temperature-dependent activation between non-capacitated and capacitated sperm.

5. The reduced temperature sensitivity observed in capacitated sperm is attributed to cleavage of the CatSper1 N terminus during capacitation. Can the authors use the observed reduction in molecular weight to estimate how many residues—particularly histidines—are removed during this cleavage?

A: The first 350 amino acids of murine CatSper1 have a predicted molecular weight of 39.9 kDa and a theoretical isoelectric point (pI) of 8.25. In our structural modeling, we used the first 332 amino acids of the N-terminal domain, corresponding to a predicted molecular weight of 37.9 kDa and a theoretical pI of 8.24. As reported by Ded et al. [5] *(Fig. 1A–K), at least the first 150 amino acids are removed during capacitation, based on the epitope recognized by their antibody. Their Figure 1K further indicates that capacitation-associated cleavage removes approximately 15–20 kDa from CatSper1, corresponding to roughly 180 amino acids from the N terminus. Although this cleavage is predicted to retain the conserved His317 residue, it removes the N-terminal arginine residues Arg6 and Arg7 and is expected to substantially disrupt the structural integrity of the N-terminal domain, thereby impairing its assembly and interactions. We have added this explanation to the revised manuscript.*

Reviewer #3: The manuscript uses AlphaFold3, among other techniques, to analyse the predicted structure of CatSper ion channels and its functional significance. The authors propose that histidine clusters "form inter-complex coupling interfaces between adjacent CatSper assemblies" and that "temperature-dependent deprotonation modulates supramolecular CatSper assembly to coordinate channel activation".
The study raises interesting hypotheses, but the manuscript suffers from several important shortcomings, detailed below:

1.The prose is often florid rather than precise. This is not merely a stylistic issue but one of clarity: the text is frequently confusing instead of straightforward. I illustrate this problem using the second part of the Abstract, but imprecise and convoluted phrasing appears throughout the text. I provide only a few examples here, as it's not the reviewer' responsibility to rewrite the manuscript to make it publishable and readable. Unfortunately, the absence of line numbers makes it unnecessarily difficult to document these issues.
"Its activation is tightly restricted to defined physiological contexts, including intracellular alkalinization, membrane depolarization, and elevated temperature." Several expressions in that sentence are unclear: What does 'tightly restricted' mean in this context? What constitutes a 'defined physiological context'? Do the authors simply mean that CatSper is gated by pH, voltage, and temperature? If so, they should state this in simple, explicit terms.
"…uncover a mechanism for temperature and pH integration." Integration into what, exactly? Do the authors intend to describe the mechanism that makes CatSper both pH-sensitive and temperature-sensitive, or a specific cellular mechanism that ntegrates the respective contributions of temperature and pH to channel activation? As written, the sentence is ambiguous.
„evolutionary hotspot". The term 'evolutionary hotspot' is not self-explanatory in this context and should be defined? Is it a mutational hotspot, recombination hotspot, biodiversity hotspot, hotspot of diversification etc.?
"N-terminal length and histidine enrichment scale with species-specific fertilization temperatures". Do the authors mean 'correlate with', instead of 'scale with'? If not, they should define precisely what type of scaling they have in mind.

"adaptive tuning of physicochemical sensitivity." The meaning if 'adaptive tuning' compared with simple 'tuning' is not clear? Likewise, physicochemical sensitivity is vague or opaque? Do the authors refer specifically to sensitivity to temperature and/or pH?
"histidine clusters form inter-complex coupling interfaces between adjacent CatSper assemblies". The distinction between complex and assembly needs to be defined. What exactly is meant by ' coupling interface' in structural or functional terms?
"dominant voltage-sensing module". What distinguishes a 'dominant' voltage sensor from a voltage-sensing module in general? The term should be deleted or replaced with more standard language.
"temperature-dependent histidine deprotonation modulates supramolecular CatSper assembly to coordinate channel activation." What, specifically, does 'modulates CatSper assembly' mean here? Which structural transitions or interactions are proposed? What is meant by 'coordination of channel activation'?
This reviewer understands that this is an Abstract with limited space. Nonetheless, the authors should invest some efforts to be es concrete and precise as possible.

A: Thank you for these thoughtful comments. We agree that several parts of the Abstract could be made more precise. We have therefore revised the Abstract throughout to improve clarity, simplify the wording where possible, and define terminology more explicitly while maintaining a concise presentation.

2. The statement„…In certain lineages—including birds—the channel has been lost…" is unclear. Do the authors claim that all birds lack CatSper or that CatSper has been lost in some bird species? The former meaning would not be correct. A clearer example of loss of CatSper would arguably be fish.

A: CatSper has been lost in avian species as has been previously reported (PMID: 18974790). In chicken, the CatSper gene cluster has degenerated into pseudogenes, preventing formation of a functional CatSper complex. Interestingly, CatSper has not been lost in all teleost lineages; for example, salmon retain CatSper genes (see Table 1). Per Cai and Clapham (PMID: 18974790): "…CatSper sequences might have been degenerated in chick genomes, with short fragments of coding sequences still present". We have revised the text to clarify that CatSper loss has been documented in avian lineages rather than implying that every bird species has been individually examined, and we now explicitly mention fish as an additional example of lineage-specific retention and loss.

3. Although Figs. 1a, c, and d are suggestive, the claimed correlation is not convincing, when the numerous exceptions are taken into account. The authors seem to recognize this problem by mentioning the mole rat as an exception, just to discard the case as special and irrelevant. For example, in Fig. 1a, corals are placed at the cold end of the temperature bar near 70 C; however, corals typically inhabit marine environments between approximately 25 and 350 C. Moreover, fish occupy habitats ranging from tropical to arctic waters. Therefore, being mostly external fertilizers, the 'fertilisation temperature' is known. Finally, did the authors consider external vs. internal fertilizers? The source and definition of 'fertilization temperature' should be clearly specified?
The fundamental problem with this analysis is that the enormous spread in the data; it's not a statistical spread but a general spread: species that fertilise at 36-400 C have N-termini ranging from 50 to 500 aa residues. Conversely, species with N-termini of roughly 400 aa, fertilise in environments with temperatures between 8 and 400 C. As a result, the proposed relationship between N-terminal length, histidine content, and 'fertilisation temperature' appears weak and heavily confounded by exceptions. In a similar vein, the model that follows from this, namely temperature-dependent modulation of His protonation that might organize CatSper activation, is neither credible nor supported by experimental data.

A: We appreciate the reviewer's concerns and have revised the manuscript to address them. Specifically, we expanded the comparative analysis of the intrinsically disordered region (IDR) of CatSper1 by classifying orthologs into three distinct categories and systematically comparing fertilization temperature, histidine enrichment, hydrophobicity, and additional sequence parameters. This expanded analysis demonstrates that histidine enrichment is the most consistent feature associated with fertilization temperature across taxa.

To address concerns regarding exceptions, we have expanded the Discussion to explain why the mole rats may represent biological outliers, given their unique subterranean habitat, altered thermoregulation, and distinctive reproductive physiology. We have also removed the temperature bar from Fig. 1a to eliminate potential confusion regarding fertilization temperatures in invertebrates. The sources and definitions of fertilization temperatures were explicitly provided in Table 1, together with appropriate references. It must be noted, that Bayesian regression analysis (Fig. S2) supports a positive posterior association between fertilization temperature and CatSper1 N-terminal length while appropriately accounting for uncertainty in the data. Additionally, we re-analyzed Figure 1 to show power law behavior (see now Figures 1e and S2b). We plotted the observable, the fertilization temperatures along the ordinate and the evolutionary controlled variable i.e., the lengths of CatSper NTD along the abscissa. After performing linear regression analysis on 32 internally fertilizing species with fertilization temperatures around 30 °C and above, Pearson correlation analysis demonstrated a significant positive correlation between the variables ($r = 0.675$, $n = 32$, $P < 0.0001$), corresponding to an R^2 of 0.456 (Fig. 1e).

Taken together, the revised manuscript incorporates additional unbiased IDR analyses, clearer definitions of fertilization temperature, improved data presentation, and complementary statistical analyses. These additions strengthen the evidence that fertilization temperature is associated with evolutionary variation in CatSper1 sequence properties, particularly histidine enrichment and N-terminal length. While we acknowledge that biological systems exhibit substantial variability and that no single parameter perfectly predicts fertilization temperature across all species, the overall evolutionary trend remains robust and is supported by multiple independent analyses.

4. Sperm used for Ca2+ fluorescence measurements were treated differently from those used for electrophysiology. In one protocol, sperm were capacitated for 60-90 min; in the other, capacitation occurred during swim-up for only 20 min. It is questionable whether 20 min are sufficient for full capacitation of sperm and for removal of the N-terminal end. The literature generally recommends longer capacitation periods (up to 3 h) rather than shorter ones. In any case, sperm used in different experiments were capacitated under different conditions.

A: There appears to be a misunderstanding regarding the capacitation protocol used for the calcium imaging experiments. In fact, all experiments were performed under comparable capacitating conditions, with murine sperm exposed to capacitating media <u>for more than 60 minutes</u>, a duration sufficient for CatSper1 N-terminal processing, consistent with previous studies. The "20 min" mentioned in the original manuscript refers only to the initial swim-up step. This is followed by a 60–90 min incubation during Fura-2AM loading, which also occurs under capacitating conditions. Thus, the total capacitation time for sperm used in calcium imaging was 80–110 min. We have clarified this in the Methods and Results sections to describe this protocol more clearly and avoid ambiguity.
It is also important to distinguish between murine and human sperm capacitation. The prolonged capacitation periods of up to 3 h apply primarily to human sperm, whereas murine sperm, which were used throughout this study, typically achieve full capacitation within 60 min and are routinely used for IVF within this time window. Extended capacitation beyond this period results in CatSper1 N-terminal processing and is associated with reduced fertilization competence, consistent with our findings that prolonged capacitation impairs sperm function following N-terminal cleavage.

Accordingly, the sperm used for both electrophysiological recordings and imaging underwent sufficient capacitation for CatSper1 N-terminal processing, and we have clarified the experimental procedures in the revised manuscript.

5. Several temperature-sensitive channels and receptors have been proposed in sperm, including ThermoTRP channels (which are also Ca2+-permeable), CatSper, and the visual pigment rhodopsin. The corresponding author has previously proposed TRPV4 as a thermosensor. As long as the individual contributions of these various thermosensors to Ca2+-signalling and fertilisation remain unknown or unclear, the strong conclusions drawn in this manuscript are largely unwarranted.

A: We have addressed this in our recent publication (Swain et al, Nature Communication, 2025)[6], demonstrating that while TRPV4 might be present in mouse and is indeed present in human sperm, its contribution to overall temperature response is a magnitude smaller in comparison to CatSper.

6. Fig.4c shows that the amplitude of the fluorescence Ca2+ signals increases with both temperature (bar graph) and time (lower axis) in non-capacitated sperm. It remains entirely unclear which other temperature-sensitive channels in sperm contribute to the Ca2+ current or fluorescence signals under these conditions. By contrast, in capacitated sperm, the temperature-related signals are smaller. The authors interpret this result to mean that the N-terminus of CatSper1 is cleaved during capacitation and that the truncated CatSper becomes temperature-insensitive. However, they do not demonstrate N-terminal cleavage in their own experiments and instead rely on the work of Ded, JJ Chung et al. (2020). Ded et al., however, used capacitation times between 90 min and 4 h, which differ substantially from those used here in some experiments.
A: The concern regarding capacitation duration is addressed in our response to Comment #4. As clarified there, the total capacitation time for the calcium imaging experiments was 80–110 min, which is comparable to the time frame in which CatSper1 N-terminal processing has been reported by Ded et al. and others.

Regarding the source of the temperature-dependent Ca^{2+} signal, our experiments using CatSper-deficient sperm demonstrate that the vast majority of the temperature-evoked Ca^{2+} response is mediated by the CatSper channel. While we cannot exclude minor contributions from other temperature-sensitive pathways, the residual signal observed in CatSper-deficient sperm is minimal, indicating that CatSper is the predominant mediator of the temperature-dependent Ca^{2+} influx under our experimental conditions.

Our interpretation that the reduced temperature sensitivity following capacitation reflects CatSper1 N-terminal processing is therefore based on the combination of (i) our functional data, (ii) the established dependence of the response on CatSper, and (iii) previous studies demonstrating N-terminal cleavage after comparable capacitation periods.

7. „Together, these findings support the conclusion that temperature gating is critical for maintaining CatSper channels organized within CatSpermasomes in a highly ordered and synchronized array, enabling their concerted opening. Disruption of this domain uncouples channels from the ordered CatSpermasome lattice, leading to asynchronous or inefficient channel opening. Loss of concerted gating reduces effective channel conductance and limits CaÇ⁺ influx."
These statements reflect either a misconception about ion channels in general, and CatSper in particular, or imprecise language. CatSper is gated by changes in pH, voltage, progesterone (in humans) and possibly temperature. At any given time, most of these parameters are constant, and a change in one parameter is sufficient to open the channel. The term 'concerted' is, therefore, misleading. If membrane voltage changes, all channels in the membrane experience that change. If the channel is activated by a puff of progesterone, all channels will be exposed to the same progesterone concentration within the mixing time (and assuming homogenous mixing). If CatSper is activated by a step increase in pHi, all channels sense the same pH (assuming rapid, millisecond equilibration along the flagellum from the site where the pH change is imposed). In none of these

scenarios is a special concerted action among channel complexes or among various stimuli required. Finally, the manuscript does not clarify the mechanisms of 'integration', i.e., how simultaneous rapid changes in two stimuli (say temperature and pH) are translated into conformational changes in an allosteric protein, like CatSper.

A: We appreciate the reviewer's comment and agree that the term "concerted" could be misinterpreted as implying classical allosteric coupling between channel molecules. This was not our intended meaning, and we have therefore revised the wording throughout the manuscript. Our hypothesis is not that neighboring CatSper channels require one another to sense voltage, pH, or temperature. Rather, our data suggests that efficient physiological activation of CatSper requires the simultaneous presence of alkaline intracellular pH, physiological membrane depolarization, and elevated temperature. As demonstrated experimentally, none of these stimuli alone produces maximal channel activation under physiological conditions. We have specifically mentioned this in the Discussion: "In addition to regulation by pH and temperature, CatSper activity is strongly modulated by membrane voltage, such that progressively greater depolarization is required to open the channel under acidic conditions [4, 6-9]. Conversely, at warmer temperatures, CatSper opens at less depolarized potentials, whereas acidic pH completely abolishes temperature-dependent gating. This interdependence between temperature, pH, and voltage suggests a shared structural basis for polymodal regulation."

In other words, for example, at intracellular pH 7.4, CatSper reaches substantial activation at elevated temperatures (V1/2= -21mV for divalent cations[6]), whereas at 24°C, at the same pH, a substantially greater unphysiological membrane depolarization (V1/2= +45mV[6]) is required to achieve comparable activation. Likewise, elevated temperature alone cannot efficiently activate CatSper at acidic pH[6]. Thus, maximal channel activity results from the combined action of these physiological stimuli. We further propose that the supramolecular organization of CatSper arrays promotes synchronized activation of neighboring channel complexes, thereby generating the large macroscopic CatSper currents observed experimentally. This model does not imply that one channel gates another directly, but rather that inter-complex interactions facilitate coordinated activation within the native CatSper array.

To avoid ambiguity, we have replaced the term concerted opening with synchronized activation throughout the manuscript and have clarified that this represents a mechanistic model supported by our structural and functional data.

Based on estimated single-channel conductance (500fS–1pS)[10], generation of the experimentally observed macroscopic CatSper currents (approximately 50–100 pA) requires the simultaneous opening of a large number of channels. We now discuss this quantitative argument in the revised manuscript.

8. In general, the manuscript contains too many speculations and inferences that are not directly supported by data. In its present form, the work appears premature. Interactions between CatSper complexes in the zig-zag rows should be demonstrated using structural techniques rather than inferred solely from simulations. In addition, temperature-induced deprotonation of His residues must be shown experimentally. The functional significance of temperature sensors in sperm remains unclear and controversial, and too many thermosensors have been proposed without definitive evidence.

A: We appreciate the reviewer's concern regarding the distinction between experimentally supported findings and the mechanistic model proposed in this study. Throughout the revised manuscript, we have taken care to more clearly distinguish our experimental observations from the hypotheses and interpretations that they support.

This manuscript was submitted as a PLOS Biology Discovery Report, a format intended for important mechanistic discoveries and novel biological insights that may not yet constitute a fully complete mechanistic characterization. In this context, we believe our combination of comparative

evolutionary analyses, AlphaFold3-based structural modeling, electrophysiology, calcium imaging, and additional IDR analyses provides a strong foundation for the mechanistic model proposed here.

We agree that direct structural visualization of inter-complex interactions in the open state and experimental determination of the protonation state of individual histidine residues would provide valuable additional evidence. However, such experiments are not currently technically feasible. CatSper can presently be studied only in its native supramolecular organization within sperm, and resolving conformational changes together with residue-specific protonation states in situ remains beyond the capabilities of current structural methods. We have therefore explicitly acknowledged these limitations in the revised Discussion and have carefully revised the manuscript to present these mechanisms as a model supported by the available experimental and computational evidence rather than as directly demonstrated structural events.

1. Eggert-Kruse W, Kohler A, Rohr G, Runnebaum B. The pH as an important determinant of sperm-mucus interaction. Fertil Steril. 1993;59(3):617-28. PubMed PMID: 8458467.
2. Maas DH, Stein B, Metzger H. PO2 and pH measurements within the rabbit oviduct following tubal microsurgery: reanastomosis of previously dissected tubes. Advances in experimental medicine and biology. 1984;169:561-70. PubMed PMID: 6731111.
3. Maas DH, Storey BT, Mastroianni L, Jr. Hydrogen ion and carbon dioxide content of the oviductal fluid of the rhesus monkey (Macaca mulatta). Fertil Steril. 1977;28(9):981-5. PubMed PMID: 19307.
4. Lishko PV, Kirichok Y, Ren D, Navarro B, Chung JJ, Clapham DE. The control of male fertility by spermatozoan ion channels. Annu Rev Physiol. 2012;74:453-75. Epub 2011/10/25. doi: 10.1146/annurev-physiol-020911-153258. PubMed PMID: 22017176.
5. Ded L, Hwang JY, Miki K, Shi HF, Chung JJ. 3D in situ imaging of the female reproductive tract reveals molecular signatures of fertilizing spermatozoa in mice. Elife. 2020;9. Epub 20201020. doi: 10.7554/eLife.62043. PubMed PMID: 33078708; PMCID: PMC7707823.
6. Swain DK, Vergara C, Castro-Arnau J, Lishko PV. The essential calcium channel of sperm CatSper is temperature-gated. Nat Commun. 2025;16(1):3657. Epub 20250417. doi: 10.1038/s41467-025-58824-0. PubMed PMID: 40246904; PMCID: PMC12006431.
7. Kirichok Y, Navarro B, Clapham DE. Whole-cell patch-clamp measurements of spermatozoa reveal an alkaline-activated Ca2+ channel. Nature. 2006;439(7077):737-40. Epub 2006/02/10. doi: nature04417 [pii]

10.1038/nature04417. PubMed PMID: 16467839.
8. Lishko PV, Botchkina IL, Kirichok Y. Progesterone Activates the Principal Ca2+ Channel of Human Sperm. Nature. 2011;471:387-91. Epub 16 March 2011. doi: 10.1038/nature09767.
9. Lishko P, Clapham DE, Navarro B, Kirichok Y. Sperm patch-clamp. Methods in enzymology. 2013;525:59-83. Epub 2013/03/26. doi: 10.1016/B978-0-12-397944-5.00004-3. PubMed PMID: 23522465.
10. Niu Q, Lishko PV. Ion Channels as Gatekeepers of Fertility: From Uterine Kir7.1 to Sperm CatSper. Physiology (Bethesda). 2026. Epub 20260609. doi: 10.1152/physiol.00015.2026. PubMed PMID: 42262737; PMCID: PMC13341003.
