# Supplementary material for: A histidine-mediated supramolecular mechanism links temperature and pH activation of the sperm channel CatSper
Source: PLoS Biol. 2026 Oct 9;24(10):e3003776. doi: 10.1371/journal.pbio.3003776 (PMC13674862; doi:10.1371/journal.pbio.3003776)
Supplement: Supplementary file 2 [file pbio.3003776.s010.docx]

The manuscript uses AlphaFold3 to analyse the predicted structure of CatSper ion channels. The authors propose that histidine clusters “form inter-complex coupling interfaces between adjacent CatSper assemblies” and that “temperature-dependent deprotonation modulates supramolecular CatSper assembly to coordinate channel activation”.

The study raises interesting hypotheses, but the manuscript suffers from several important shortcomings, detailed below:

1.The prose is often florid rather than precise. This is not merely a stylistic issue but one of clarity: the text is frequently confusing instead of straightforward. I illustrate this problem using the second part of the Abstract, but imprecise and convoluted phrasing appears throughout the text. I provide only a few examples here, as it’s not the reviewer’ responsibility to rewrite the manuscript to make it publishable and readable. Unfortunately, the absence of line numbers makes it unnecessarily difficult to document these issues.

“*Its activation is tightly restricted to defined physiological contexts, including intracellular alkalinization, membrane depolarization, and elevated temperature.*” Several expressions in that sentence are unclear: What does ‘tightly restricted’ mean in this context? What constitutes a ‘defined physiological context’? Do the authors simply mean that CatSper is gated by pH, voltage, and temperature? If so, they should state this in simple, explicit terms.

*“…uncover a mechanism for temperature and pH integration.”* Integration into what, exactly? Do the authors intend to describe the mechanism that makes CatSper both pH-sensitive and temperature-sensitive, or a specific cellular mechanism that ntegrates the respective contributions of temperature and pH to channel activation? As written, the sentence is ambiguous.

*„evolutionary hotspot“*. The term ‘evolutionary hotspot’ is not self-explanatory in this context and should be defined? Is it a mutational hotspot, recombination hotspot, biodiversity hotspot, hotspot of diversification etc.?

*“N-terminal length and histidine enrichment scale with species-specific fertilization temperatures”*. Do the authors mean ‘correlate with’, instead of ‘scale with’? If not, they should define precisely what type of scaling they have in mind.

*“adaptive tuning of physicochemical sensitivity.”* The meaning if ‘adaptive tuning’ compared with simple ‘tuning’ is not clear? Likewise, physicochemical sensitivity is vague or opaque? Do the authors refer specifically to sensitivity to temperature and/or pH?

*“histidine clusters form inter-complex coupling interfaces between adjacent CatSper assemblies”.* The distinction between complex and assembly needs to be defined. What exactly is meant by ‘ coupling interface’ in structural or functional terms?

*“dominant voltage-sensing module”.* What distinguishes a ‘dominant’ voltage sensor from a voltage-sensing module in general? The term should be deleted or replaced with more standard language.

*“temperature-dependent histidine deprotonation modulates supramolecular CatSper assembly to coordinate channel activation.”* What, specifically, does ‘modulates CatSper assembly’ mean here? Which structural transitions or interactions are proposed? What is meant by ‘coordination of channel activation’?

This reviewer understands that this is an Abstract with limited space. Nonetheless, the authors should invest some efforts to be es concrete and precise as possible.

2. The statement*„…In certain lineages—including birds—the channel has been lost…”* is unclear. Do the authors claim that all birds lack CatSper or that CatSper has been lost in some bird species? The former meaning would not be correct. A clearer example of loss of CatSper would arguably be fish.

3. Although Figs. 1a, c, and d are suggestive, the claimed correlation is not convincing, when the numerous exceptions are taken into account. The authors seem to recognize this problem by mentioning the mole rat as an exception, just to discard the case as special and irrelevant. For example, in Fig. 1a, corals are placed at the cold end of the temperature bar near 7^0^ C; however, corals typically inhabit marine environments between approximately 25 and 35^0^ C. Moreover, fish occupy habitats ranging from tropical to arctic waters. Therefore, being mostly external fertilizers, the ‘fertilisation temperature’ is known. Finally, did the authors consider external vs. internal fertilizers? The source and definition of ‘fertilization temperature’ should be clearly specified?

The fundamental problem with this analysis is that the enormous spread in the data; it’s not a statistical spread but a general spread: species that fertilise at 36-40^0^ C have N-termini ranging from 50 to 500 aa residues. Conversely, species with N-termini of roughly 400 aa, fertilise in environments with temperatures between 8 and 40^0^ C. As a result, the proposed relationship between N-terminal length, histidine content, and ‘fertilisation temperature’ appears weak and heavily confounded by exceptions. In a similar vein, the model that follows from this, namely temperature-dependent modulation of His protonation that might organize CatSper activation, is neither credible nor supported by experimental data.

4. Sperm used for Ca^2+^ fluorescence measurements were treated differently from those used for electrophysiology. In one protocol, sperm were capacitated for 60-90 min; in the other, capacitation occurred during swim-up for only 20 min. It is questionable whether 20 min are sufficient for full capacitation of sperm and for removal of the N-terminal end. The literature generally recommends longer capacitation periods (up to 3 h) rather than shorter ones. In any case, sperm used in different experiments were capacitated under different conditions.

5. Several temperature-sensitive channels and receptors have been proposed in sperm, including ThermoTRP channels (which are also Ca^2+^-permeable), CatSper, and the visual pigment rhodopsin. The corresponding author has previously proposed TRPV4 as a thermosensor. As long as the individual contributions of these various thermosensors to Ca^2+^-signalling and fertilisation remain unknown or unclear, the strong conclusions drawn in this manuscript are largely unwarranted.

6. Fig.4c shows that the amplitude of the fluorescence Ca^2+^ signals increases with both temperature (bar graph) and time (lower axis) in non-capacitated sperm. It remains entirely unclear which other temperature-sensitive channels in sperm contribute to the Ca^2+^ current or fluorescence signals under these conditions. By contrast, in capacitated sperm, the temperature-related signals are smaller. The authors interpret this result to mean that the N-terminus of CatSper1 is cleaved during capacitation and that the truncated CatSper becomes temperature-insensitive. However, they do not demonstrate N-terminal cleavage in their own experiments and instead rely on the work of Ded, JJ Chung et al. (2020). Ded et al., however, used capacitation times between 90 min and 4 h, which differ substantially from those used here in some experiments.

7. *„Together, these findings support the conclusion that temperature gating is critical for maintaining CatSper channels organized within CatSpermasomes in a highly ordered and synchronized array, enabling their concerted opening. Disruption of this domain uncouples channels from the ordered CatSpermasome lattice, leading to asynchronous or inefficient channel opening. Loss of concerted gating reduces effective channel conductance and limits CaÇ⁺ influx.”*

These statements reflect either a misconception about ion channels in general, and CatSper in particular, or imprecise language. CatSper is gated by changes in pH, voltage, progesterone (in humans) and possibly temperature. At any given time, most of these parameters are constant, and a change in one parameter is sufficient to open the channel. The term ‘concerted’ is, therefore, misleading. If membrane voltage changes, all channels in the membrane experience that change. If the channel is activated by a puff of progesterone, all channels will be exposed to the same progesterone concentration within the mixing time (and assuming homogenous mixing). If CatSper is activated by a step increase in pH_i_, all channels sense the same pH (assuming rapid, millisecond equilibration along the flagellum from the site where the pH change is imposed). In none of these scenarios is a special concerted action among channel complexes or among various stimuli required. Finally, the manuscript does not clarify the mechanisms of ‘integration’, i.e., how simultaneous rapid changes in two stimuli (say temperature and pH) are translated into conformational changes in an allosteric protein, like CatSper.

8. In general, the manuscript contains too many speculations and inferences that are not directly supported by data. In its present form, the work appears premature. Interactions between CatSper complexes in the zig-zag rows should be demonstrated using structural techniques rather than inferred solely from simulations. In addition, temperature-induced deprotonation of His residues must be shown experimentally. The functional significance of temperature sensors in sperm remains unclear and controversial, and too many thermosensors have been proposed without definitive evidence.
