# Supplementary material for: A histidine-mediated supramolecular mechanism links temperature and pH activation of the sperm channel CatSper
Source: PLoS Biol. 2026 Oct 9;24(10):e3003776. doi: 10.1371/journal.pbio.3003776 (PMC13674862; doi:10.1371/journal.pbio.3003776)
Supplement: Supplementary file 3 [file pbio.3003776.s013.docx]

Dear *PLOS Biology* Editors,

We would like to again thank the Editors and Reviewers for their supportive, constructive, and helpful feedback, as well as for the time and effort they have devoted to evaluating our manuscript.

In response to the remaining suggestion, we have revised the manuscript to provide a clearer explanation of why prolonged capacitation conditions can result in reduced CatSper channel activity, particularly in the murine system. We clarify this in the Introduction. We also clarify further in the Result section, that the optimal duration of capacitation differs among mammalian species and that mouse sperm undergo capacitation over a substantially shorter time scale than sperm from larger mammals, such as bovine or human sperm.

Our responses are provided below in italics. Please note that the figure order has been updated to reflect the requested change to shorten number of main Figures to 4. The remaining revisions are indicated by blue in the marked-up version of the manuscript.

**REVIEWER COMMENTS**

Reviewer #1: After some confusion about the correct version of the paper in the website and figure changes, I reviewed the R2 version and found that authors have answered all my questions. Readers new to the area may be confused as to why CatSper currents are decreased after 60 minute capacitation, when they would expect them to increase in order to increase motility. This could be explained in the introduction, along with a definition of capacitation. I also believe the authors have answered Reviewer 2 and 3's questions.

*A: We thank the Reviewer for this helpful suggestion. We have revised the Introduction to include a definition of capacitation and to clarify why CatSper activity can decline following prolonged capacitation. Specifically, we now explain that, in the murine system, capacitation occurs over a substantially shorter time scale than in larger mammals, such as bovine and humans. Thus, although capacitation is required to progressively prime sperm for optimal function, prolonged incubation beyond the optimal capacitation window can lead to changes in the CatSper complex that reduce its functional activity. We hope that this clarification will help readers unfamiliar with the field better understand why CatSper currents decline following extended capacitation conditions in mouse sperm.*

Reviewer #2: The authors have satisfactorily addressed my previous comments, which were minor. I have no additional suggestion.

*A: We sincerely thank the Reviewer for the supportive evaluation and are pleased that we were able to satisfactorily address the previous comments.*
