# Supplementary material for: A histidine-mediated supramolecular mechanism links temperature and pH activation of the sperm channel CatSper
Source: PLoS Biol. 2026 Oct 9;24(10):e3003776. doi: 10.1371/journal.pbio.3003776 (PMC13674862; doi:10.1371/journal.pbio.3003776)
Supplement: Peer review history

# Editor report: Decision Letter 0

**DOI:** 10.1371/journal.pbio.3003776.r001

**Authors:** Richard Hodge

7 Apr 2026

Dear Polina,

Thank you for submitting your manuscript entitled "Structural Insights into the Integration of Temperature and pH by Sperm Calcium Channel CatSper" for consideration as a Discovery Report by PLOS Biology. Please accept my sincere apologies for the delay in getting back to you as we consulted with an academic editor about your submission.

Your manuscript has now been evaluated by the PLOS Biology editorial staff, as well as by an academic editor with relevant expertise, and I am writing to let you know that we would like to send your submission out for external peer review.

However, before we can send your manuscript to reviewers, we need you to complete your submission by providing the metadata that is required for full assessment. To this end, please login to Editorial Manager where you will find the paper in the 'Submissions Needing Revisions' folder on your homepage. Please click 'Revise Submission' from the Action Links and complete all additional questions in the submission questionnaire.

Once your full submission is complete, your paper will undergo a series of checks in preparation for peer review. After your manuscript has passed the checks it will be sent out for review. To provide the metadata for your submission, please Login to Editorial Manager (https://www.editorialmanager.com/pbiology) within two working days, i.e. by Apr 09 2026 11:59PM.

If your manuscript has been previously peer-reviewed at another journal, PLOS Biology is willing to work with those reviews in order to avoid re-starting the process. Submission of the previous reviews is entirely optional and our ability to use them effectively will depend on the willingness of the previous journal to confirm the content of the reports and share the reviewer identities. Please note that we reserve the right to invite additional reviewers if we consider that additional/independent reviewers are needed, although we aim to avoid this as far as possible. In our experience, working with previous reviews does save time.

If you would like us to consider previous reviewer reports, please edit your cover letter to let us know and include the name of the journal where the work was previously considered and the manuscript ID it was given. In addition, please upload a response to the reviews as a 'Prior Peer Review' file type, which should include the reports in full and a point-by-point reply detailing how you have or plan to address the reviewers' concerns.

During the process of completing your manuscript submission, you will be invited to opt-in to posting your pre-review manuscript as a bioRxiv preprint. Visit http://journals.plos.org/plosbiology/s/preprints for full details. If you consent to posting your current manuscript as a preprint, please upload a single Preprint PDF.

Feel free to email us at plosbiology@plos.org if you have any queries relating to your submission.

Kind regards,

Richard

Richard Hodge, PhD

Senior Editor, PLOS Biology

rhodge@plos.org

PLOS

Empowering researchers to transform science

Carlyle House, Carlyle Road, Cambridge, CB4 3DN, United Kingdom

California (U.S.) corporation #C2354500, based in San Francisco

# Peer review: Decision Letter 1

**DOI:** 10.1371/journal.pbio.3003776.r002

**Authors:** Richard Hodge

23 May 2026

Dear Polina,

Thank you for your patience while your manuscript "Structural Insights into the Integration of Temperature and pH by Sperm Calcium Channel CatSper" was peer-reviewed at PLOS Biology. Please accept my sincere apologies for the delays that you have experienced during the peer review process. Your manuscript has now been evaluated by the PLOS Biology editors, an Academic Editor with relevant expertise, and by three independent reviewers.

In light of the reviews, which you will find at the end of this email, we would like to invite you to revise the work to thoroughly address the reviewers' reports.

As you will see below, Reviewer’s #1 and #2 are generally positive and think the paper is interesting, but they provide several comments to improve the presentation, reporting and the strength of discussion. On the other hand, Reviewer #3 is more negative and raises concerns that the language used in the manuscript text is unclear and imprecise at several points. In addition, the reviewer raises important technical concerns with the experiments and that the central model of temperature-dependent modulation of His protonation and CatSper activation is not fully supported by the data. After discussions with the Academic Editor, we ask that you provide a detailed response to the comments provided by Reviewer #3 and temper the conclusions regarding the role of temperature gating alone.

Given the extent of revision needed, we cannot make a decision about publication until we have seen the revised manuscript and your response to the reviewers' comments. Your revised manuscript is likely to be sent for further evaluation by all or a subset of the reviewers.

In addition to these revisions, you will need to complete some formatting changes, which you will receive in a follow up email. A member of our team will be in touch with a set of requests shortly.

We expect to receive your revised manuscript within 3 months. Please email us (plosbiology@plos.org) if you have any questions or concerns, or would like to request an extension.

At this stage, your manuscript remains formally under active consideration at our journal; please notify us by email if you do not intend to submit a revision so that we may withdraw it.

**IMPORTANT - SUBMITTING YOUR REVISION**

Your revisions should address the specific points made by each reviewer. Please submit the following files along with your revised manuscript:

1. A 'Response to Reviewers' file - this should detail your responses to the editorial requests, present a point-by-point response to all of the reviewers' comments, and indicate the changes made to the manuscript.

*NOTE: In your point-by-point response to the reviewers, please provide the full context of each review. Do not selectively quote paragraphs or sentences to reply to. The entire set of reviewer comments should be present in full and each specific point should be responded to individually, point by point.

You should also cite any additional relevant literature that has been published since the original submission and mention any additional citations in your response.

2. In addition to a clean copy of the manuscript, please also upload a 'track-changes' version of your manuscript that specifies the edits made. This should be uploaded as a "Revised Article with Changes Highlighted" file type.

*Re-submission Checklist*

When you are ready to resubmit your revised manuscript, please refer to this re-submission checklist: https://plos.io/Biology_Checklist

To submit a revised version of your manuscript, please go to https://www.editorialmanager.com/pbiology/ and log in as an Author. Click the link labelled 'Submissions Needing Revision' where you will find your submission record.

Please make sure to read the following important policies and guidelines while preparing your revision:

*Published Peer Review*

Please note while forming your response, if your article is accepted, you may have the opportunity to make the peer review history publicly available. The record will include editor decision letters (with reviews) and your responses to reviewer comments. If eligible, we will contact you to opt in or out. Please see here for more details:

https://blogs.plos.org/plos/2019/05/plos-journals-now-open-for-published-peer-review/

*PLOS Data Policy*

Please note that as a condition of publication PLOS' data policy (http://journals.plos.org/plosbiology/s/data-availability) requires that you make available all data used to draw the conclusions arrived at in your manuscript. If you have not already done so, you must include any data used in your manuscript either in appropriate repositories, within the body of the manuscript, or as supporting information (N.B. this includes any numerical values that were used to generate graphs, histograms etc.). For an example see here: http://www.plosbiology.org/article/info%3Adoi%2F10.1371%2Fjournal.pbio.1001908#s5

*Blot and Gel Data Policy*

We require the original, uncropped and minimally adjusted images supporting all blot and gel results reported in an article's figures or Supporting Information files. We will require these files before a manuscript can be accepted so please prepare them now, if you have not already uploaded them. Please carefully read our guidelines for how to prepare and upload this data: https://journals.plos.org/plosbiology/s/figures#loc-blot-and-gel-reporting-requirements

*Protocols deposition*

To enhance the reproducibility of your results, we recommend that if applicable you deposit your laboratory protocols in protocols.io, where a protocol can be assigned its own identifier (DOI) such that it can be cited independently in the future. Additionally, PLOS ONE offers an option for publishing peer-reviewed Lab Protocol articles, which describe protocols hosted on protocols.io. Read more information on sharing protocols at https://plos.org/protocols?utm_medium=editorial-email&utm_source=authorletters&utm_campaign=protocols

Thank you again for your submission to our journal. We hope that our editorial process has been constructive thus far, and we welcome your feedback at any time. Please don't hesitate to contact us if you have any questions or comments.

Best regards,

Richard

Richard Hodge, PhD

Senior Editor, PLOS Biology

rhodge@plos.org

------------------------------------

REVIEWS:

Reviewer #1: The paper by Zhao et al is a fascinating examination of the evolutionary mechanism for CatSper channel complex sensitivity to pH and temperature, enabling sperm capacitation when the sperm encounter the alkaline pH and body temperature of the vaginal fornix after intercourse. CatSper arrays, containing ~ 15 distinct subunits, are probably the most complex ion channels in nature, reflecting substantial refinement and strong evolutionary pressure. Part of its unusual nature is the length of its N terminus in higher animals, which is enriched with histidine residues (17% in mouse CatSper1 NTD, similar to other histidine rich proteins that sense pH). The N-terminal domain (NTD) of CatSper1 varies significantly across species, correlating with fertilization temperature. Histidine-arginine interactions may stabilize inter-CatSper complexes via cation-π interactions, sensitive to pH and temperature.

The experimental data appear strong. Electrophysiology shows reduced temperature-dependent currents after NTD degradation. Calcium imaging indicates capacitated sperm lose temperature gating. Proteolytic removal of the N terminus correlates with loss of temperature sensitivity. Histidine residues within the NTD mediate pH-dependent assembly via π-π and cation-π interactions, stabilized at alkaline pH with deprotonated histidines. The experimental, structural and AlphaFold data together support the hypothesis that elevated temperature and alkaline pH causes histidine deprotonation, stabilizing inter-complex coupling.

In my opinion, the paper requires no new experimental data. My suggestions are mainly for discussion unless the data are available in the literature.

1.Perhaps this is not known, but it would be interesting to the measured pericervical pH during fertilization, particularly for the mole rat. I suspect the data is not known, but correlation of pH across species, like temperature, would be interesting. The pH variation may account from some of the deviations in the temperature plots of various species.

2.The lipid droplet idea is interesting, but it would be good to reinforce with what is known about lipid droplet formation conditions and state the lipid composition of the sperm membrane containing the CatSper complex compared to other cell membranes.

Minor: Grammar

'partial degradation of N-terminal domain' should be 'partial degradation of the N-terminal domain'

', feature truncated NTD barely reaching' should be ', feature truncated NTDs barely reaching'

"The modeled distances between these arginines and neighboring histidines (3.7-4.3 Å) fall within the range compatible with stabilizing cation-π interactions (3-6 A)"- include the part in parentheses

Reviewer #2: In this interesting manuscript, the authors address a long-standing question that has intrigued researchers for over 25 years: why CatSper1, a sperm restricted ion channel protein that is fundamentally important for sperm motility and male fertility, contains so many histidine residues in its N terminus. Using evolutionary analyses, the authors make the intriguing observation that histidine content increases during metazoan evolution, correlating with increasing fertilization temperature.

Using AlphaFold3 based modeling, the authors identify a potential interaction between the histidine rich N terminus and the canonical gating machinery of the channel, suggesting a mechanism for temperature sensitive channel gating mediated by the histidine rich domain. Taking advantage of previous findings showing that part of the CatSper1 N terminus is cleaved during sperm capacitation, the authors compare the temperature sensitivity of CatSper using patch clamp recordings and Ca²⁺ imaging in capacitated versus non capacitated sperm. Consistent with their hypothesis, capacitated sperm—which presumably lack part of the N terminus—exhibit reduced temperature sensitivity in channel activation.

Overall, these findings are very interesting and provide potential answers to a long standing biological problem. The authors' approach is both unique and appropriate. Importantly, this question could not have been effectively addressed using alternative strategies, given the intrinsically disordered nature of the N terminus (which precludes high resolution structural determination) and the inability to reconstitute CatSper channels in heterologous expression systems despite decades of effort. As discussed by the authors, the results presented here are also likely to stimulate future studies in the field.

Specific comments:

1.In the structural models, it would be helpful to draw a membrane and clearly indicate extracellular and intracellular spaces.

2.In the Introduction, the statement "The cation channel of sperm (CatSper) is a sperm specific, calcium ion channel that is essential for male fertility across the evolutionary tree" may be overstated. The cited literature (refs. 1-15) appears to demonstrate essential roles for CatSper primarily in humans and mice. Is there evidence that CatSper is essential for male fertility in additional species across the evolutionary tree?

3.In Fig. 3c and d, the traces labeled "non capacitated CatSper1⁺/⁺ (WT)" in the two panels appear to be the same. If these traces are replotted, this should be clearly stated in the figure legend.

4.In Fig. 4b, while it is clear that capacitated sperm exhibit smaller currents across all temperatures, the temperature dependence of channel activation is less apparent. To better illustrate temperature sensitivity, it would be helpful to also plot the ratio of currents measured at 41 °C to those measured at 24 °C.

5.The reduced temperature sensitivity observed in capacitated sperm is attributed to cleavage of the CatSper1 N terminus during capacitation. Can the authors use the observed reduction in molecular weight to estimate how many residues—particularly histidines—are removed during this cleavage?

Reviewer #3: The manuscript uses AlphaFold3, among other techniques, to analyse the predicted structure of CatSper ion channels and its functional significance. The authors propose that histidine clusters "form inter-complex coupling interfaces between adjacent CatSper assemblies" and that "temperature-dependent deprotonation modulates supramolecular CatSper assembly to coordinate channel activation".

The study raises interesting hypotheses, but the manuscript suffers from several important shortcomings, detailed below:

1.The prose is often florid rather than precise. This is not merely a stylistic issue but one of clarity: the text is frequently confusing instead of straightforward. I illustrate this problem using the second part of the Abstract, but imprecise and convoluted phrasing appears throughout the text. I provide only a few examples here, as it's not the reviewer' responsibility to rewrite the manuscript to make it publishable and readable. Unfortunately, the absence of line numbers makes it unnecessarily difficult to document these issues.

"Its activation is tightly restricted to defined physiological contexts, including intracellular alkalinization, membrane depolarization, and elevated temperature." Several expressions in that sentence are unclear: What does 'tightly restricted' mean in this context? What constitutes a 'defined physiological context'? Do the authors simply mean that CatSper is gated by pH, voltage, and temperature? If so, they should state this in simple, explicit terms.

"…uncover a mechanism for temperature and pH integration." Integration into what, exactly? Do the authors intend to describe the mechanism that makes CatSper both pH-sensitive and temperature-sensitive, or a specific cellular mechanism that ntegrates the respective contributions of temperature and pH to channel activation? As written, the sentence is ambiguous.

„evolutionary hotspot". The term 'evolutionary hotspot' is not self-explanatory in this context and should be defined? Is it a mutational hotspot, recombination hotspot, biodiversity hotspot, hotspot of diversification etc.?

"N-terminal length and histidine enrichment scale with species-specific fertilization temperatures". Do the authors mean 'correlate with', instead of 'scale with'? If not, they should define precisely what type of scaling they have in mind.

"adaptive tuning of physicochemical sensitivity." The meaning if 'adaptive tuning' compared with simple 'tuning' is not clear? Likewise, physicochemical sensitivity is vague or opaque? Do the authors refer specifically to sensitivity to temperature and/or pH?

"histidine clusters form inter-complex coupling interfaces between adjacent CatSper assemblies". The distinction between complex and assembly needs to be defined. What exactly is meant by ' coupling interface' in structural or functional terms?

"dominant voltage-sensing module". What distinguishes a 'dominant' voltage sensor from a voltage-sensing module in general? The term should be deleted or replaced with more standard language.

"temperature-dependent histidine deprotonation modulates supramolecular CatSper assembly to coordinate channel activation." What, specifically, does 'modulates CatSper assembly' mean here? Which structural transitions or interactions are proposed? What is meant by 'coordination of channel activation'?

This reviewer understands that this is an Abstract with limited space. Nonetheless, the authors should invest some efforts to be es concrete and precise as possible.

2. The statement„…In certain lineages—including birds—the channel has been lost…" is unclear. Do the authors claim that all birds lack CatSper or that CatSper has been lost in some bird species? The former meaning would not be correct. A clearer example of loss of CatSper would arguably be fish.

3. Although Figs. 1a, c, and d are suggestive, the claimed correlation is not convincing, when the numerous exceptions are taken into account. The authors seem to recognize this problem by mentioning the mole rat as an exception, just to discard the case as special and irrelevant. For example, in Fig. 1a, corals are placed at the cold end of the temperature bar near 70 C; however, corals typically inhabit marine environments between approximately 25 and 350 C. Moreover, fish occupy habitats ranging from tropical to arctic waters. Therefore, being mostly external fertilizers, the 'fertilisation temperature' is known. Finally, did the authors consider external vs. internal fertilizers? The source and definition of 'fertilization temperature' should be clearly specified?

The fundamental problem with this analysis is that the enormous spread in the data; it's not a statistical spread but a general spread: species that fertilise at 36-400 C have N-termini ranging from 50 to 500 aa residues. Conversely, species with N-termini of roughly 400 aa, fertilise in environments with temperatures between 8 and 400 C. As a result, the proposed relationship between N-terminal length, histidine content, and 'fertilisation temperature' appears weak and heavily confounded by exceptions. In a similar vein, the model that follows from this, namely temperature-dependent modulation of His protonation that might organize CatSper activation, is neither credible nor supported by experimental data.

4. Sperm used for Ca2+ fluorescence measurements were treated differently from those used for electrophysiology. In one protocol, sperm were capacitated for 60-90 min; in the other, capacitation occurred during swim-up for only 20 min. It is questionable whether 20 min are sufficient for full capacitation of sperm and for removal of the N-terminal end. The literature generally recommends longer capacitation periods (up to 3 h) rather than shorter ones. In any case, sperm used in different experiments were capacitated under different conditions.

5. Several temperature-sensitive channels and receptors have been proposed in sperm, including ThermoTRP channels (which are also Ca2+-permeable), CatSper, and the visual pigment rhodopsin. The corresponding author has previously proposed TRPV4 as a thermosensor. As long as the individual contributions of these various thermosensors to Ca2+-signalling and fertilisation remain unknown or unclear, the strong conclusions drawn in this manuscript are largely unwarranted.

6. Fig.4c shows that the amplitude of the fluorescence Ca2+ signals increases with both temperature (bar graph) and time (lower axis) in non-capacitated sperm. It remains entirely unclear which other temperature-sensitive channels in sperm contribute to the Ca2+ current or fluorescence signals under these conditions. By contrast, in capacitated sperm, the temperature-related signals are smaller. The authors interpret this result to mean that the N-terminus of CatSper1 is cleaved during capacitation and that the truncated CatSper becomes temperature-insensitive. However, they do not demonstrate N-terminal cleavage in their own experiments and instead rely on the work of Ded, JJ Chung et al. (2020). Ded et al., however, used capacitation times between 90 min and 4 h, which differ substantially from those used here in some experiments.

7. „Together, these findings support the conclusion that temperature gating is critical for maintaining CatSper channels organized within CatSpermasomes in a highly ordered and synchronized array, enabling their concerted opening. Disruption of this domain uncouples channels from the ordered CatSpermasome lattice, leading to asynchronous or inefficient channel opening. Loss of concerted gating reduces effective channel conductance and limits CaÇ⁺ influx."

These statements reflect either a misconception about ion channels in general, and CatSper in particular, or imprecise language. CatSper is gated by changes in pH, voltage, progesterone (in humans) and possibly temperature. At any given time, most of these parameters are constant, and a change in one parameter is sufficient to open the channel. The term 'concerted' is, therefore, misleading. If membrane voltage changes, all channels in the membrane experience that change. If the channel is activated by a puff of progesterone, all channels will be exposed to the same progesterone concentration within the mixing time (and assuming homogenous mixing). If CatSper is activated by a step increase in pHi, all channels sense the same pH (assuming rapid, millisecond equilibration along the flagellum from the site where the pH change is imposed). In none of these scenarios is a special concerted action among channel complexes or among various stimuli required. Finally, the manuscript does not clarify the mechanisms of 'integration', i.e., how simultaneous rapid changes in two stimuli (say temperature and pH) are translated into conformational changes in an allosteric protein, like CatSper.

8. In general, the manuscript contains too many speculations and inferences that are not directly supported by data. In its present form, the work appears premature. Interactions between CatSper complexes in the zig-zag rows should be demonstrated using structural techniques rather than inferred solely from simulations. In addition, temperature-induced deprotonation of His residues must be shown experimentally. The functional significance of temperature sensors in sperm remains unclear and controversial, and too many thermosensors have been proposed without definitive evidence.

Attachment Submitted filename: Review_PLoSBiology_Lishko.docx

# Author comment: Author response to Decision Letter 2

**DOI:** 10.1371/journal.pbio.3003776.r003

30 Jul 2026

Attachment Submitted filename: Response to Reviewers Zhao et al 72626.pdf

# Peer review: Decision Letter 2

**DOI:** 10.1371/journal.pbio.3003776.r004

**Authors:** Richard Hodge

1 Sep 2026

Dear Polina,

Thank you for your patience while we considered your revised manuscript "Structural Insights into the Integration of Temperature and pH by Sperm Calcium Channel CatSper" for publication as a Discovery Report at PLOS Biology. This revised version of your manuscript has been evaluated by the PLOS Biology editors, the Academic Editor and two of the original reviewers.

Based on the reviews, I am pleased to say that we are likely to accept this manuscript for publication, provided you satisfactorily address the remaining points raised by Reviewer #1. In addition, Please also make sure to address the following data and other policy-related requests that I have provided below (A-G):

(A) We are considering your manuscript as a Discovery Report, which have a maximum of 4 main figures. At this stage, I would be grateful if the number of main figures could be reduced down to 4, either by combining two of the main figures or by moving one to the supplementary.

(B) We routinely suggest changes to titles to ensure maximum accessibility for a broad, non-specialist readership. In this case, we would suggest the following edit to the title for additional information. Please ensure you change both the manuscript file and the online submission system, as they need to match for final acceptance:

“A histidine-mediated supramolecular mechanism links temperature and pH activation of the sperm channel CatSper”

(C) You may be aware of the PLOS Data Policy, which requires that all data be made available without restriction: http://journals.plos.org/plosbiology/s/data-availability. For more information, please also see this editorial: http://dx.doi.org/10.1371/journal.pbio.1001797

Note that we do not require all raw data. Rather, we ask that all individual quantitative observations that underlie the data summarized in the figures and results of your paper be made available in one of the following forms:

-Supplementary files (e.g., excel). Please ensure that all data files are uploaded as 'Supporting Information' and are invariably referred to (in the manuscript, figure legends, and the Description field when uploading your files) using the following format verbatim: S1 Data, S2 Data, etc. Multiple panels of a single or even several figures can be included as multiple sheets in one excel file that is saved using exactly the following convention: S1_Data.xlsx (using an underscore).

-Deposition in a publicly available repository. Please also provide the accession code or a reviewer link so that we may view your data before publication.

Regardless of the method selected, please ensure that you provide the individual numerical values that underlie the summary data displayed in the following figure panels as they are essential for readers to assess your analysis and to reproduce it:

Figure 1B-D, 2A-B, 4B-D, S1A-B, S2A-B, S3A-C

NOTE: the numerical data provided should include all replicates AND the way in which the plotted mean and errors were derived (it should not present only the mean/average values).

(D) Please also ensure that each of the relevant figure legends in your manuscript include information on *WHERE THE UNDERLYING DATA CAN BE FOUND*, and ensure your supplemental data file/s has a legend.

(E) Please ensure that your Data Statement in the submission system accurately describes where your data can be found and is in final format, as it will be published as written there.

(F) Per journal policy, if you have generated any custom code during the course of this investigation, please make it available without restrictions. Please ensure that the code is sufficiently well documented and reusable, and that your Data Statement in the Editorial Manager submission system accurately describes where your code can be found. More information on our Code Policy, what and how to share can be found here: https://journals.plos.org/plosbiology/s/code-availability

Please note that we cannot accept sole deposition of code in GitHub, as this could be changed after publication. However, you can archive this version of your publicly available GitHub code to Zenodo. Once you do this, it will generate a DOI number, which you will need to provide in the Data Accessibility Statement (you are welcome to also provide the GitHub access information). See the process for doing this here: https://docs.github.com/en/repositories/archiving-a-github-repository/referencing-and-citing-content

(G) Please note that per journal policy, the model system/species studied should be clearly stated in the abstract of your manuscript.

------------------------------------------------------------------------

As you address these items, please take this last chance to review your reference list to ensure that it is complete and correct. If you have cited papers that have been retracted, please include the rationale for doing so in the manuscript text, or remove these references and replace them with relevant current references. Any changes to the reference list should be mentioned in the cover letter that accompanies your revised manuscript.

In addition to these revisions, you may need to complete some formatting changes, which you will receive in a follow up email. A member of our team will be in touch with a set of requests shortly. If you do not receive a separate email within a few days, please assume that checks have been completed, and no additional changes are required.

We expect to receive your revised manuscript within two weeks.

To submit your revision, please go to https://www.editorialmanager.com/pbiology/ and log in as an Author. Click the link labelled 'Submissions Needing Revision' to find your submission record. Your revised submission must include the following:

- a cover letter that should detail your responses to any editorial requests, if applicable, and whether changes have been made to the reference list

- a Response to Reviewers file that provides a detailed response to the reviewers' comments (if applicable, if not applicable please do not delete your existing 'Response to Reviewers' file.)

- a track-changes file indicating any changes that you have made to the manuscript.

NOTE: If Supporting Information files are included with your article, note that these are not copyedited and will be published as they are submitted. Please ensure that these files are legible and of high quality (at least 300 dpi) in an easily accessible file format. For this reason, please be aware that any references listed in an SI file will not be indexed. For more information, see our Supporting Information guidelines:

https://journals.plos.org/plosbiology/s/supporting-information

*Published Peer Review History*

Please note that you may have the opportunity to make the peer review history publicly available. The record will include editor decision letters (with reviews) and your responses to reviewer comments. If eligible, we will contact you to opt in or out. Please see here for more details:

https://plos.org/published-peer-review-history/

*Press*

Should you, your institution's press office or the journal office choose to press release your paper, please ensure you have opted out of Early Article Posting on the submission form. We ask that you notify us as soon as possible if you or your institution is planning to press release the article.

*Protocols deposition*

To enhance the reproducibility of your results, we recommend that if applicable you deposit your laboratory protocols in protocols.io, where a protocol can be assigned its own identifier (DOI) such that it can be cited independently in the future. Additionally, PLOS ONE offers an option for publishing peer-reviewed Lab Protocol articles, which describe protocols hosted on protocols.io. Read more information on sharing protocols at https://plos.org/protocols?utm_medium=editorial-email&utm_source=authorletters&utm_campaign=protocols

Please do not hesitate to contact me should you have any questions.

Best wishes,

Richard

Richard Hodge, PhD

Senior Editor, PLOS Biology

rhodge@plos.org

------------------------------------------------------------------------

Reviewer remarks:

Reviewer #1: After some confusion about the correct version of the paper in the website and figure changes, I reviewed the R2 version and found that authors have answered all my questions. Readers new to the area may be confused as to why CatSper currents are decreased after 60 minute capacitation, when they would expect them to increase in order to increase motility. This could be explained in the introduction, along with a definition of capacitation.

I also believe the authors have answered Reviewer 2 and 3's questions.

Reviewer #2: The authors have satisfactorily addressed my previous comments, which were minor. I have no additional suggestion.

# Author comment: Author response to Decision Letter 3

**DOI:** 10.1371/journal.pbio.3003776.r005

22 Sep 2026

Attachment Submitted filename: Response to Reviewers R2 Zhao et al 91026.docx

# Editor report: Decision Letter 3

**DOI:** 10.1371/journal.pbio.3003776.r006

**Authors:** Richard Hodge

23 Sep 2026

Dear Polina,

On behalf of my colleagues and the Academic Editor, Raimund Dutzler, I am pleased to say that we can accept your manuscript for publication, provided you address any remaining formatting and reporting issues. These will be detailed in an email you should receive within 2-3 business days from our colleagues in the journal operations team; no action is required from you until then. Please note that we will not be able to formally accept your manuscript and schedule it for publication until you have completed any requested changes.

**Please note that I neglected to ask you to include a competing interest statement in the previous round given your role on the PLOS Biology Editorial Board. So, I have included the following sentence in the online submission form on your behalf, according to our policies:

"I have read the journal’s policy and the authors of this manuscript have the following competing interests: P.L is a member of PLOS Biology’s Editorial Board."

Please take a minute to log into Editorial Manager at http://www.editorialmanager.com/pbiology/, click the "Update My Information" link at the top of the page, and update your user information to ensure an efficient production process.

PRESS

We frequently collaborate with press offices. If your institution or institutions have a press office, please notify them about your upcoming paper at this point, to enable them to help maximise its impact. If the press office is planning to promote your findings, we would be grateful if they could coordinate with biologypress@plos.org. If you have previously opted in to the early version process, we ask that you notify us immediately of any press plans so that we may opt out on your behalf.

We also ask that you take this opportunity to read our Embargo Policy regarding the discussion, promotion and media coverage of work that is yet to be published by PLOS. As your manuscript is not yet published, it is bound by the conditions of our Embargo Policy. Please be aware that this policy is in place both to ensure that any press coverage of your article is fully substantiated and to provide a direct link between such coverage and the published work. For full details of our Embargo Policy, please visit http://www.plos.org/about/media-inquiries/embargo-policy/.

Thank you again for choosing PLOS Biology for publication and supporting Open Access publishing. We look forward to publishing your study.

Best wishes,

Richard

Richard Hodge, PhD

Senior Editor, PLOS Biology

rhodge@plos.org

PLOS

Empowering researchers to transform science

Carlyle House, Carlyle Road, Cambridge, CB4 3DN, United Kingdom

California (U.S.) corporation #C2354500, based in San Francisco
